# Membrane platform protein PulF of the *Klebsiella* type II secretion system forms a trimeric ion channel essential for endopilus assembly and protein secretion

**DOI:** 10.1128/mbio.01423-23

**Published:** 2023-12-08

**Authors:** Ingrid Guilvout, Firdaus Samsudin, Roland G. Huber, Peter J. Bond, Benjamin Bardiaux, Olivera Francetic

**Affiliations:** 1 Institut Pasteur, Université Paris Cité, CNRS UMR 3528, Biochemistry of Macromolecular Interactions Unit, Paris, France; 2 Bioinformatics Institute (A-STAR), Singapore, Singapore; 3 Department of Biological Sciences, National University of Singapore, Singapore, Singapore; 4 Institut Pasteur, Université Paris Cité, CNRS UMR 3528, Structural Bioinformatics Unit, Paris, France; 5 Institut Pasteur, Université Paris Cité, CNRS UMR 3528, Bacterial Transmembrane Systems Unit, Paris, France; Max-Planck-Institut fur terrestrische Mikrobiologie, Marburg, Germany

**Keywords:** type II secretion system, type IV pili, type IV filaments, ion channel, cardiolipin, residue coevolution, AlphaFold2, ion motive force

## Abstract

Type IV pili and type II secretion systems (T2SS) are crucial for bacterial adaptation, virulence, and environmental impact. A common mechanism underlying their multiple functions involves assembly of dynamic plasma membrane-anchored filaments—the (endo)pili. The cytoplasmic ATPase motor GspE/PilB is thought to energize pilus assembly *via* the membrane assembly platform protein GspF/PilC, but platform protein structure and its molecular role remain elusive. Here, to dissect the GspF/PilC architecture and mechanism, we generated all-atom models of the *Klebsiella* T2SS platform protein PulF in different oligomeric states. Comprehensive modeling, molecular dynamics (MD) simulations, cysteine crosslinking, and biochemical analyses support the trimeric state of PulF. In the trimer, the transmembrane segment TMS2 and the nonessential cytoplasmic N-domain are peripherally located, while TMS1 and TMS3 form a 6-helix bundle delineating a central transmembrane channel. Polar and proline residue pairs in these segments, conserved in all GspF/PilC homologs, define the channel constriction that can accommodate sodium ions or protons. Remarkably, obstructing this channel via Cys crosslinking abolished endopilus assembly and protein secretion, shedding light on previous findings showing that dissipating the membrane potential with ionophores reversibly abolished T2SS function. The trimeric PulF shows an excellent fit with the PulE ATPase hexamer, building a complex with structural similarities to the V-ATPase. MD simulations of PulF inserted in an *Escherichia coli* membrane model reveal strong binding and enrichment in cardiolipin, the phospholipid known to stimulate ATPase activity of GspE/PilB. We propose that GspF/PilC cooperates with the ATPase to energize (endo)pilus assembly using the ion motive force.

**IMPORTANCE:**

Type IV pili and type II secretion systems are members of the widespread type IV filament (T4F) superfamily of nanomachines that assemble dynamic and versatile surface fibers in archaea and bacteria. The assembly and retraction of T4 filaments with diverse surface properties and functions require the plasma membrane platform proteins of the GspF/PilC superfamily. Generally considered dimeric, platform proteins are thought to function as passive transmitters of the mechanical energy generated by the ATPase motor, to somehow promote insertion of pilin subunits into the nascent pilus fibers. Here, we generate and experimentally validate structural predictions that support the trimeric state of a platform protein PulF from a type II secretion system. The PulF trimers form selective proton or sodium channels which might energize pilus assembly using the membrane potential. The conservation of the channel sequence and structural features implies a common mechanism for all T4F assembly systems. We propose a model of the oligomeric PulF—PulE ATPase complex that provides an essential framework to investigate and understand the pilus assembly mechanism.

## INTRODUCTION

Prokaryotes of archaeal and bacterial kingdoms assemble dynamic surface fibers called type IV filaments (T4F), which mediate diverse cellular functions, including adherence to substrates, motility, and transport of macromolecules across their cell envelope (1). Prominent members of this superfamily include archaeal flagella and pili (2), bacterial type IV pili (T4P) (3–5), and type II secretion systems (T2SS) (6, 7). T4F assembly nanomachines may have emerged early in the common ancestor of archaea and bacteria, to spread and diversify among prokaryotes (8). Remarkably, they were recently detected in some lower eukaryotes, which harbor mitochondrial T2SS evolved from an alpha-proteobacterial ancestor of this organelle (9, 10).

**Fig 1:**
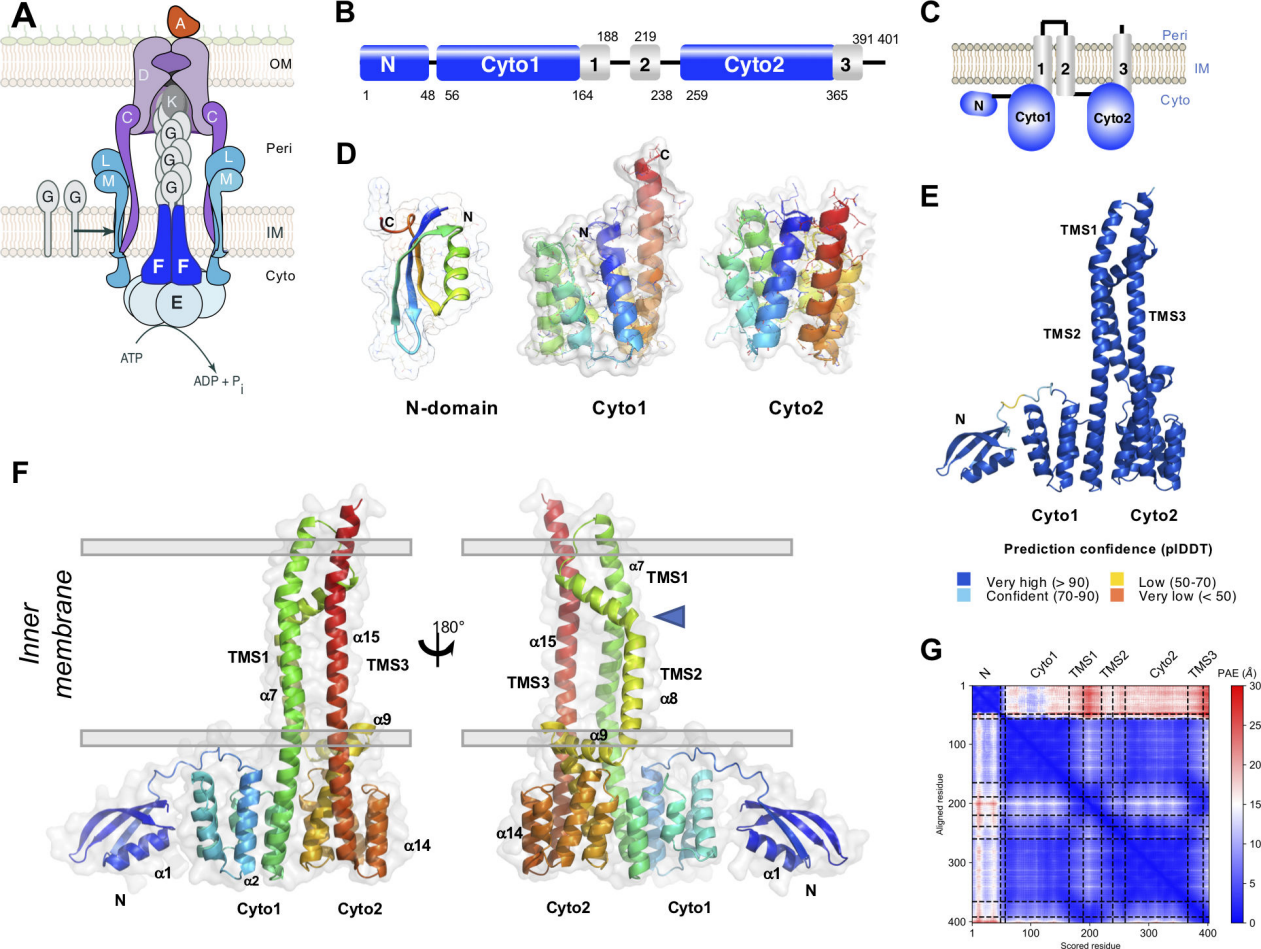
PulF domain organization. (**A**) The T2SS complex in the bacterial envelope. PulF (dark blue) is part of the assembly platform comprising the ATPase E, L, and M (light blue). The major pilin (**G**) and the tip minor pilin (**K**) are in gray. The D secretin and C complex are in purple and the substrate (**A**) in orange. IM, inner membrane; OM, outer membrane. (**B**) PulF domains in the primary sequence and (**C**) in the topology model, also predicted by MEMSAT (Fig. S1A). Domains N, Cyto1, and Cyto2 are shown in blue, TM segments in gray, and connecting regions as black lines. (**D**) Models of N, Cyto1, and Cyto2 domains generated by Robetta and coevolution analysis, shown in cartoon and transparent surface representation, rainbow-colored from the N-terminus (dark blue) to the C-terminus (red). (**E**) The AF2 model of PulF, colored by predicted local Distance Difference Test (plDDT) values, from dark blue (high confidence) to orange (very low confidence). (**F**) Side views of the AF2 PulF model shown in cartoon and transparent surface representation, rainbow-colored from the N-terminus (dark blue) to the C-terminus (red). The positions of N, Cyto1, Cyto2, TM segments, and some alpha helices are indicated. Gray lines show IM boundaries with cytoplasmic and periplasmic compartments. (**G**) Predicted aligned error (PAE) of the PulF model colored from blue (low error) to red (high error). High PAE values between the N-domain and the rest of the protein indicate that the positioning of the N-domain relative to the cytoplasmic domains is not well defined.

All these functionally diverse nanomachines share the ability to build dynamic helical filaments from pilin subunits initially embedded in the plasma membrane. While most T4F are cell surface exposed, the T2SSs form short periplasmic endopili (formerly called pseudopili) (11) that drive protein secretion (6, 7). The hexameric ATPase of the GspE/PilB family and the polytopic membrane protein of the GspF/PilC family located at the filament base are thought to promote pilin polymerization, with the aid of other proteins of the so-called inner membrane (IM) assembly platform (AP) (12) (Fig. 1A).

GspF/PilC proteins are essential components of all T4F systems studied so far, including T4P from *Neisseria gonorrhoeae* (13), *N. meningitidis* (14), *Pseudomonas aeruginosa* (15), *Myxococcus xanthus* (16), *Escherichia coli* (17), and the recently described T4P from the Gram-positive bacterium *Streptococcus sanguinis* (18). GspF homologs in T2SSs are essential for protein secretion in *Dickeya dadantii* (19), *Klebsiella oxytoca* (20), *Vibrio cholerae* (21), and *P. aeruginosa* (22). In the *K. oxytoca* T2SS, PulF is required for secretion of its substrate pullulanase (PulA) and for assembly of endopili, which can form surface fibers under conditions of moderate overexpression of T2SS-encoding genes (23). Similarly, deleting *xcpS* encoding the PulF homolog in *P. aeruginosa* T2SS blocks protein secretion and assembly of surface pili (24).

Although GspF is essential, our understanding of its structure and molecular function is limited. Sequence-based predictions and protein fusion studies (22, 25) define GspF as a polytopic IM protein, comprising three cytoplasmic domains and three transmembrane (TM) segments. X-ray crystallography provided structural information for the cytoplasmic domain Cyto1 from the *V. cholerae* EpsF with a 6-helix bundle fold (21). Structures of Cyto1 domains of PilC from *Thermus thermophilus* T4P (26) and TcpE from the *V. cholerae* toxin-coregulated pilus (27) are highly similar, indicating high conservation across these classes of T4F assembly systems. Despite several attempts, no structural information has been obtained for the N-domain, nor for the Cyto2 which shares sequence similarity and common evolutionary origin with Cyto1 (28). Analysis of the *N. meningitidis* homolog PilG by negative-stained electron microscopy (EM) resulted in a cone-shaped structure that was estimated to represent a tetramer (29). Most other studies suggest a dimeric state of GspF/PilC, including biochemical (30) and cryo-electron tomography (cryo-ET) analyses of T4P (16, 31) and T2SS (32). The Cryo-ET data support the central position of GspF/PilC in the AP complex (Fig. 1A) and, therefore, a role in transmitting the conformational changes of the cytoplasmic ATPase to the pilin subunits to energize and orchestrate pilus polymerization (4, 33). Models of how PilC dimer fits within the asymmetric cavity formed by the hexameric PilB have been proposed to explain this transmission (34). However, the lack of atomic-level information on the full-length GspF structure and interactions with its partners has precluded full mechanistic understanding of its role in filament assembly.

Given the limited insights gained from structural studies so far, here, we combined computational and biochemical approaches to generate and validate atomic models of the *Klebsiella* T2SS platform protein PulF. These models, supported by site-specific crosslinking, co-evolution, and functional analyses, reveal that PulF forms trimers which delineate a transmembrane channel crucial for T2SS function. Molecular dynamics (MD) simulation studies of PulF flexibility, interactions with model membranes, and channel properties suggest that GspF/PilC family proteins act as proton or ion channels that use membrane potential to power T4F assembly systems.

## RESULTS

### Modeling the PulF cytoplasmic domains and the full-length protein

The assembly platform protein PulF is predicted to occupy the center of the IM assembly platform complex in the *Klebsiella* T2SS (Fig. 1A). This polytopic IM protein with an N-in–C-out orientation comprises three cytoplasmic domains: N, Cyto1, and Cyto2 and three TM segments (Fig. 1B and C; Fig. S1A). The PsiPred algorithm (35) predicts an all α-helical secondary structure of PulF, except for the N-terminal domain comprising an α-helix and three β-strands (Fig. S1B). To investigate its structure–function relationship, we sought to build the complete PulF atomic model. First, we used classical approaches to model isolated domains. Since there was no homologous sequence for which a structure was solved, we generated *de novo* models of the N-domain with the Robetta server (36), which predicted a well-defined globular structure with a ββαβ fold (Fig. 1D). To ensure that the *ab initio* approach provided reliable prediction, we also used the residue co-evolution-based Gremlin algorithm (37), a template-free method to obtain spatial long-range information. Based on the predicted contacts, structural modeling yielded an N-domain structure that was virtually superimposable with the Robetta model (Fig. S2A) with a backbone root mean square deviation (RMSD) of 1.8 Å (Fig. S2B).

A model of the PulF Cyto1 domain (Fig. 1D) was generated by comparative modeling using the crystal structure of the corresponding domain from the *V. cholerae* EpsF (PDB ID 3C1Q) (21) that shares 57% sequence identity with PulF Cyto1. Although there are no structural data on Cyto2 domains, its structural similarity with Cyto1 is supported by the sequence identity levels of 28%–39% found in the GspF family (29% in *K. oxytoca* PulF) (21). It is, thus, safe to assume that Cyto1 and Cyto2 domains have a highly similar 6-helix bundle fold and we constructed a model for the PulF Cyto2 domain again using the *V. cholerae* EpsF-Cyto1 structure as a template (31% sequence identity) (Fig. 1D).

Modeling of the full-length PulF structure was performed using the machine learning-based prediction tool AlphaFold2 (AF2) (38). In the AF2 model, the N, Cyto1, and Cyto2 domains were virtually identical to their *ab initio* and homology models (Cα RMSD of 1.1 Å, 0.6 Å, and 1.2 Å, respectively), thus independently confirming the reliability of the predictions. The overall prediction confidence of the AF2 model is high (plDDT 94.8 and ptm 0.79), except in regions predicted to face the periplasm and in the flexible loop connecting the N and Cyto1 domains (Fig. 1E ; Fig. S3). The TMS1 and TMS3 are predicted to be close and run parallel to each other. The α8 helix comprising TMS2 is linked to TMS1 by a short periplasmic loop and shows a pronounced kink at the boundary with its amphiphilic segment facing the periplasm (Fig. 1F, blue triangle). The TMS2 is followed by the α9 helix, running perpendicular to α8, predicted to line the cytoplasmic face of the IM and forming, together with α8, a D-shaped arm, separating TMS2 from TMS1 and TMS3. The overall confidence reported by AF2 for the relative positioning of both cytoplasmic domains and TMS helices is high as demonstrated by the low predicted aligned error (PAE) values (Fig. 1G). However, the orientation of the N-domain with regard to the rest of the protein is uncertain with generally high PAE (Fig. 1G), consistent with the presence of an unstructured β3—α2 loop which connects the N-domain to downstream regions.

### PulF residue coevolution and conservation

**Fig 2:**
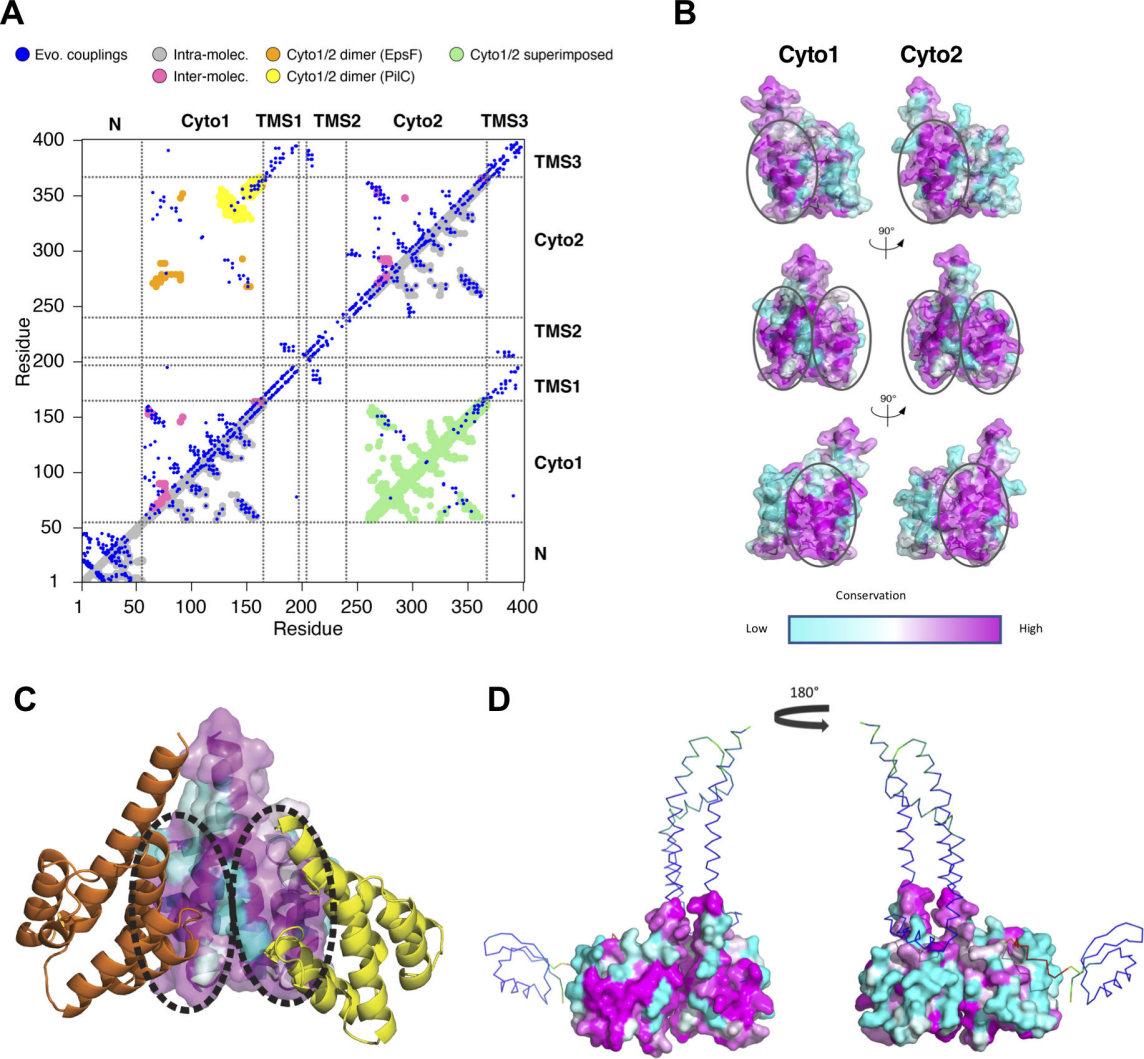
Implication of PulF domains in oligomerization. (**A**) Contact map of PulF protein from evolutionary couplings (blue), superimposed with the contacts in N-domain, Cyto1 and Cyto2 models in gray (intra, lower triangle) or pink (inter in crystal homo-dimers, upper triangle). Contacts observed in Cyto1/Cyto2 complex model based on EpsF or PilC crystal dimers are shown in orange or yellow, respectively (upper triangle). Green dots correspond to matching residues when Cyto1 and Cyto2 domains are superimposed (lower triangle). (**B**) Residue conservation on the Cyto1 and Cyto2 domains surface, color-coded from highly conserved residues (dark magenta) to the least conserved ones (dark cyan). Patches of conserved residues of Cyto1 and Cyto2 domains are circled. (**C**) Different orientations of Cyto1 domains in crystallographic dimers of EpsF (orange) and PilC (yellow). The two dimers were superimposed on one Cyto1 domain (colored by residue conservation). The conserved patches at the surface of Cyto1 are circled as in (B). (**D**) Cartoon model of PulF monomer with the space-filling representation of Cyto1 and Cyto2 domains colored according to the level of residue conservation. The conserved patches (in magenta) are surface exposed in the left view.

Based on the X-ray crystallography (21), biochemical (30), and cryo-ET (16) data, it has been generally assumed that GspF/PilC proteins form dimers (15, 34). To assess the possible domain-domain interfaces, we analyzed the co-evolution of residues across the PulF family with the Gremlin algorithm (Fig. 2A). While the N-domain showed no evolutionary contacts with other regions, possible conserved contacts were identified between the Cyto1 and Cyto2 domains (Fig. 2A, blue dots). Some of these contacts correspond to inter-monomer contacts in the crystallographic Cyto1 dimer structures (Fig. 2A, yellow and orange dots), but it remains arduous to conclude if the PulF dimer interfaces only involve Cyto1 and Cyto2 domains (Fig. 2A). One cannot exclude that these predicted contacts are an artifact of the Gremlin approach since Cyto1 and Cyto2 arise from internal gene duplication and share a similar evolutionary history and 3D fold (28). Indeed, it was shown that analysis of residue co-variance in multiple sequence alignments of pseudo-repeat proteins often incorrectly predicts contacts between repeated segments (39, 40) (Fig. 2A, green dots). It is also difficult to unambiguously distinguish intra- and inter-subunit contacts from such predictions, especially if the interface is symmetric in a homodimer with a parallel topology. Unambiguous contacts are predicted between TMS1 and TMS3, but none involving TMS2 (Fig. 2A).

Analysis of sequence conservation of surface residues with Consurf (41) revealed two conserved patches on the surface of the Cyto1 and Cyto2 domains (Fig. 2B). These patches also correspond to the two different dimer interfaces observed in the crystal structures of EpsF (21) and PilC (26) (Fig. 2C), supporting their implication in domain or oligomerization interfaces. Mapping the residue conservation on the AF2 model of PulF (Fig. 2D) shows that only one of the conserved patches at the surface of Cyto1 and Cyto2 is involved in the intra-protomer interface, while the other one is exposed (Fig. 2D). This is consistent with the oligomerization of AP proteins, also evidenced by the Bacterial Two-Hybrid (BACTH) studies of PulF (42).

### AlphaFold models of PulF oligomeric states

**Fig 3:**
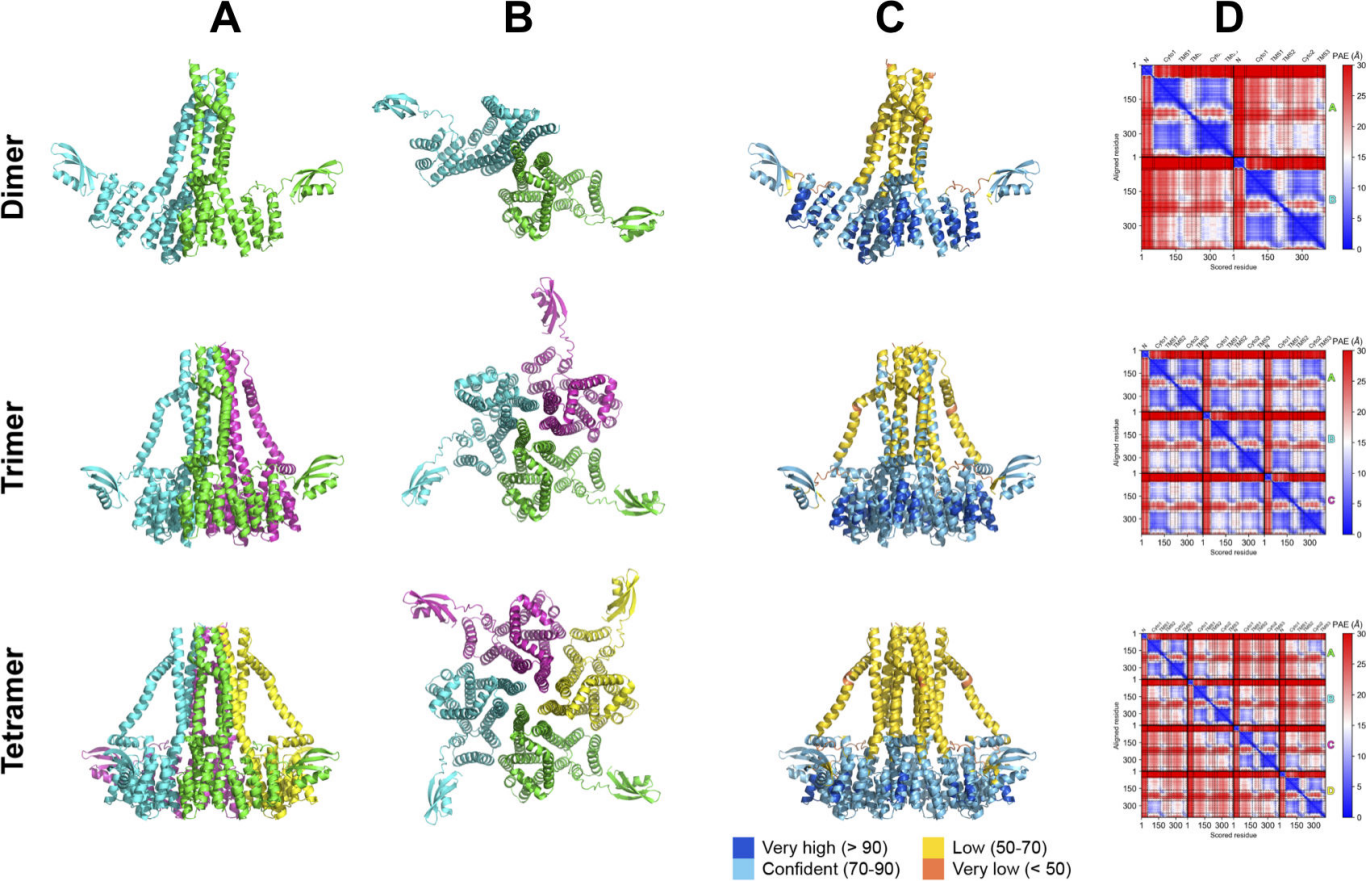
AlphaFold models of PulF oligomeric states. The best AlphaFold-multimer model for PulF as homo-dimer (top row), -trimer (middle row), or -tetramer (bottom row) are shown as cartoons. For the side (**A**) and top view (B, rotated 90°), each chain is colored differently. (C) Best models colored by predicted local Distance Difference Test (plDDT) from orange (low confidence) to blue (high confidence). The N- and cytoplasmic domains show high confidence, while the TMS display lower plDDT values. (**D**) Corresponding predicted aligned error (PAE) maps, colored from blue (low error) to red (high error). The position of each domain is labeled on top. Black lines separate the different chains in the oligomers which are labeled on the right in their respective colors. Except for the trimer model, inter-chain PAE values (off-diagonal squares) are high, reflecting a lower confidence in these interfaces.

Owing to the improved accuracy of the latest version of the AlphaFold2-Multimer dedicated to the prediction of multi-chain complexes (43), we predicted the structure of PulF in different homo-oligomeric states (Fig. 3). Using two, three, and four copies of the full-length PulF sequence, AF2-Multimer generated quasi-symmetric models of dimeric, trimeric, and tetrameric PulF with cyclic point symmetry. In each state, the inter-subunit interface involves the cytoplasmic domains and the TMS1 and TMS3 helices. However, in the dimeric PulF models, only the Cyto2 domains interact with each other. In the trimeric and tetrameric states, each Cyto1 domain has a large interaction surface with the Cyto2 domain of the neighboring protomer, thus burying the second conserved patch (Fig. 2D) while forming a ring of alternating Cyto1/Cyto2 domains (Fig. 3B). Interestingly, in these states, the long helices including the hydrophobic TMS1 and TMS3 from neighboring protomers associate in a parallel manner to form a relatively tight multi-helix bundle shielding the open ring of Cyto domains. The monomer structure is similar in oligomeric models with Cα RMSD relative to the monomeric PulF model (excluding the N-domain) of 2.3 Å, 2.4 Å, and 2.6 Å for the dimer, trimer, and tetramer models. Small reorientations of the TMS helices and cytoplasmic domains are observed to accommodate the inter-protomer interfaces. As seen for the monomer model, the position of the N-domain is still not reliably predicted in the different oligomers (Fig. 3D).

The best-scoring models for the three oligomeric states display high confidence at the residue level with plDDT values of 73.1, 76.9, and 77.1 for the dimer, trimer, and tetramer, respectively (Fig. S3). Contrary to the monomeric PulF model, the confidence is lower for the TMS helices than for the cytoplasmic domains (Fig. 3C). Compared to the dimer and the tetramer models, the model of the PulF trimer has the highest predicted TM-score (a metric that served as a proxy for the model accuracy) and the best confidence score for inter-domain contacts (Fig. S3 and S4). Even the lowest ranked AF2 model of the trimer displays a higher *iptm* (interface predicted TM-score) than the best dimer and tetramer models. In fact, the trimeric model is the only modeled oligomer where the inter-protomer PAE is significantly low (Fig. 3D), with a high number of confidently predicted contacts between cytoplasmic domains and TMS from neighboring protomers (Fig. S4 and S5B).

When comparing the evolutionary contacts with the dimer and trimer model, it appears that the trimeric model is more compatible with the contacts predicted between the Cyto1 and Cyto2 domains (Fig. S5A). The interface area is also larger for the trimer (2,945 Å^2^) and the tetramer (3,082 Å^2^) between one monomer and the rest of the complex than for the dimer (1,428 Å^2^). Taken together, AlphaFold predictions for three possible oligomeric states seem to support more favorably a trimeric assembly of PulF.

### The peripherally localized N-domain is not essential for PulF stability, function and oligomerization

**Fig 4:**
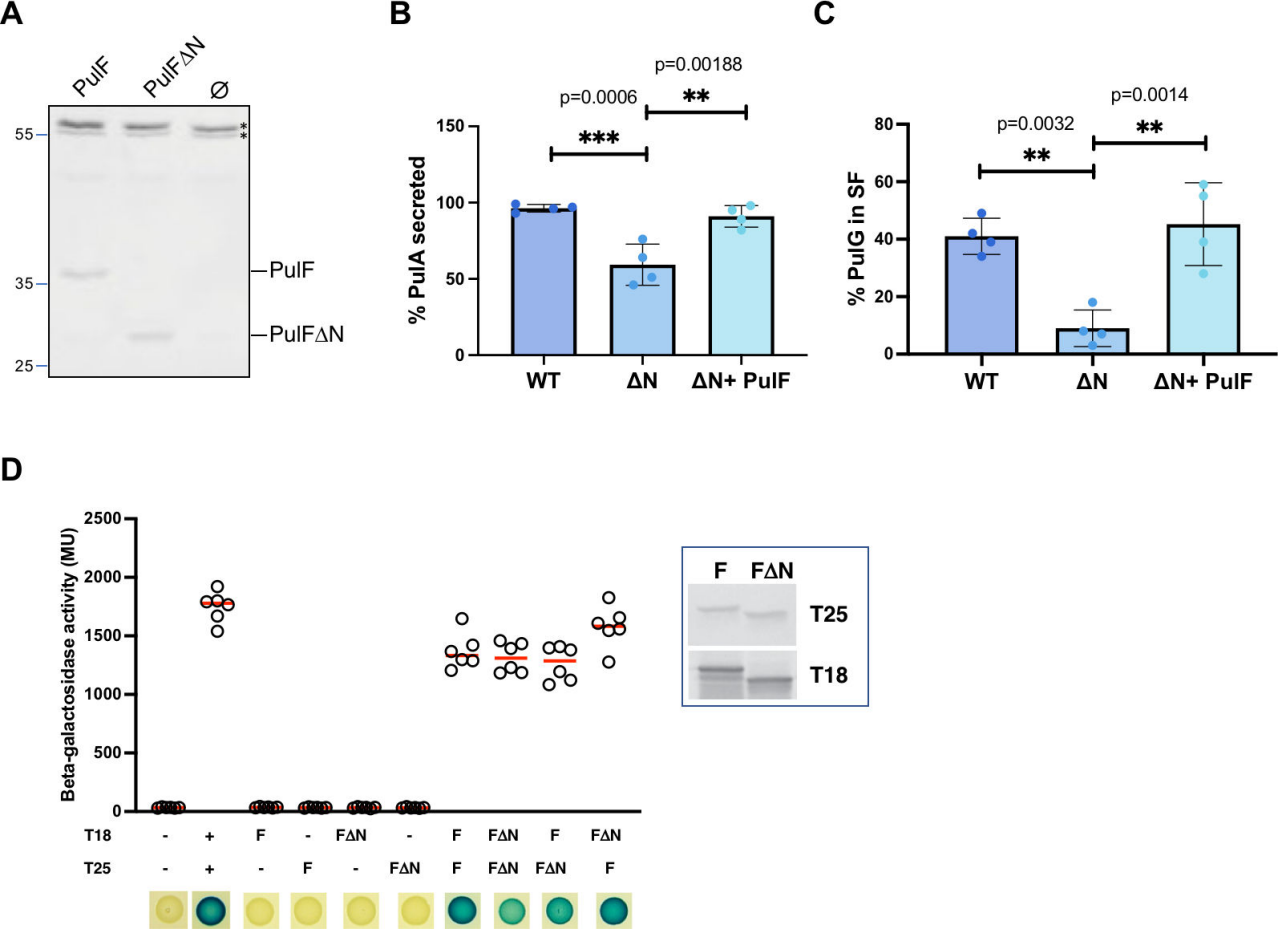
N-domain is not essential for PulF oligomerization and function. (**A**) Immunodetection of PulF in *E. coli* PAP7460 total extracts carrying pCHAP8259 (PulF), pCHAP6469 (PulFΔN), or vector pSU19 (Ø), using anti-PulF_C_ antibodies. The asterisks indicate non-specific cross-reacting bands serving as internal loading controls. (**B**) Analysis of PulA secretion and (**C**) PulG pilus assembly in the presence of PulF (WT), PulFΔN (ΔN), and PulFΔN complemented with *pulF* on pCHAP8259. Dots indicate fractions of surface-exposed PulA and PulG (*n* = 4) with mean values (bar heights). Significant differences between the mean values are indicated: ** (*P* < 0.01); *** (*P* < 0.001). The *P* values are shown on the graphs. (**D**) Bacterial two-hybrid analysis shows that the N-domain is not required for dimerization. The β-galactosidase activities of cultures producing indicated T18 and T25 derivatives shown as open circles (*n* = 8), red lines show median values. The Lac phenotypes on X-Gal IPTG plates are shown below. The inset shows protein levels of T18- and T25- PulF^WT^ and PulFΔN chimera. The signs − and + indicate negative and positive control.

In the PulF models, the N-domain is connected *via* the flexible linker to the PulF core formed by the TM segments and domains Cyto1 and Cyto2, consistent with the high PAE values and variable relative positioning of this domain. To assess the N-domain function, we generated the variant PulFΔN by deleting the *pulF* gene fragment encoding residues 2–54 in the context of the *pul* operon, in plasmid pCHAP6601. Western blot analysis with PulF_C_ antibodies showed similar levels of native PulF and PulFΔN, indicating that the N-domain is not required for protein stability (Fig. 4A). Interestingly, when analyzed by denaturing sodium dodecyl-sulfate polyacrylamide gel electrophoresis (SDS-PAGE), both proteins migrated faster than expected based on their predicted Mw (44,167 Da for PulF and 38,144 for PulFΔN), likely due to their high hydrophobicity and enhanced SDS binding. Such behavior is observed for other polytopic membrane proteins (44) including PulF homologs in T4P assembly systems (13, 45). PulFΔN variant supported PulA secretion and PulG pilus assembly albeit with significantly lower efficiency compared to native PulF (Fig. 4B and C). The functional defects were fully complemented *in trans* by the *pulF* on plasmid pCHAP8259. These results show that the core domains of PulF ensure the essential function of T2SS, while the N-domain, although non-essential, contributes significantly to its activity.

We used the bacterial two-hybrid (BACTH) assay (46) to assess the effect of N-domain deletion on PulF oligomerization. The N-terminal T18 and T25 CyaA fragments fused to the full-length, membrane-embedded PulF promote the reconstitution of adenylyl cyclase activity, as observed previoulsy (42), indicating their interaction (Fig. 4D). When the N-domain-encoding fragment was deleted in these BACTH constructs (Table S1), the PulF and PulFΔN hybrid proteins showed equally strong interaction signals in the BACTH assay (Fig. 4D) and were produced at similar levels (Fig. 4D, inset). This indicates that the N-domain is not involved in the interface, consistent with the coevolution analysis (Fig. 2A) and with the peripheral localization of the N-domain in all oligomer models (Fig. 3).

### The hydrophobic regions TMS1 and TMS3 are involved in PulF oligomerization

**Fig 5:**
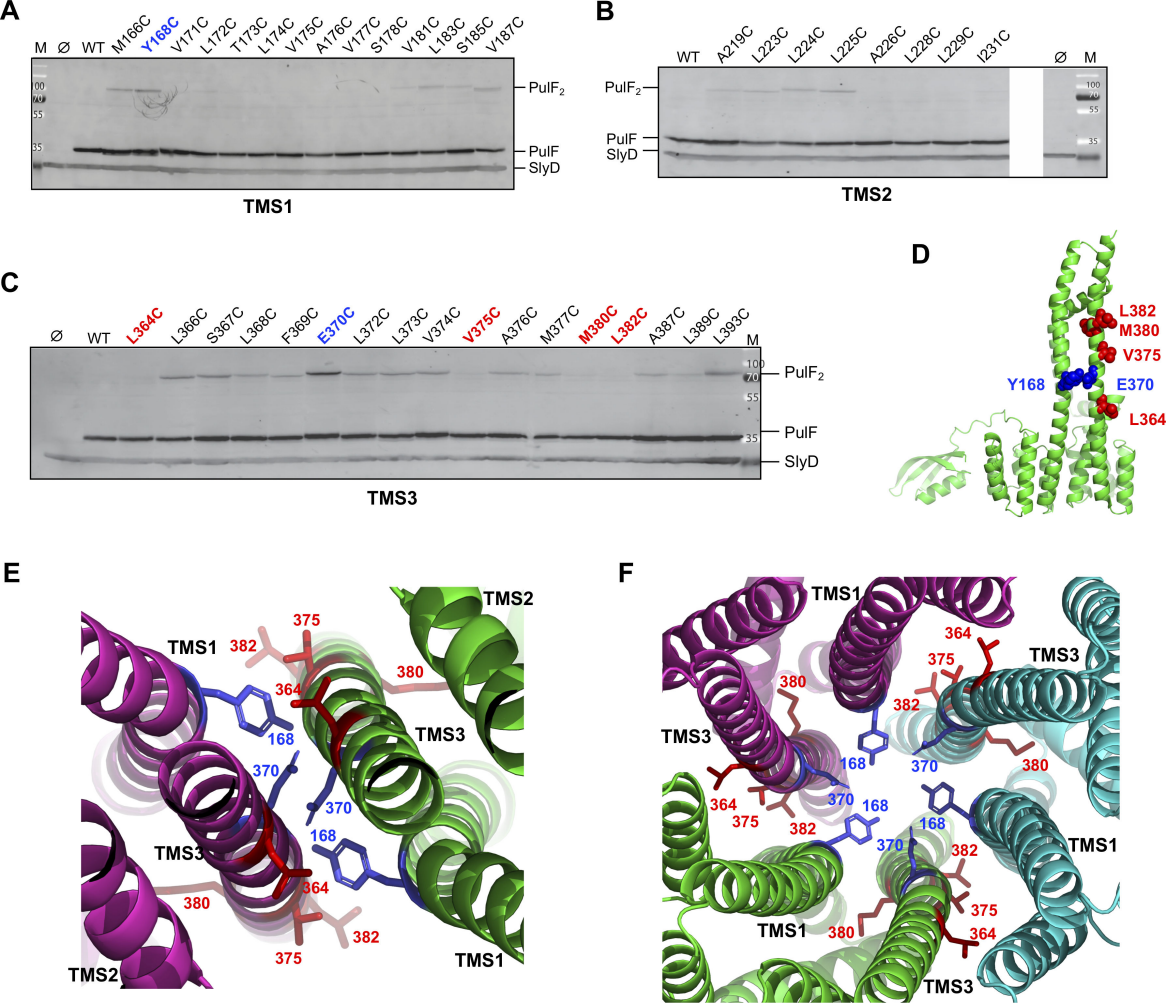
Cysteine crosslinking analysis of the PulF membrane oligomerization interface. Cys substitutions were introduced at indicated positions of PulF TM segments 1 (**A**), 2 (**B**), and 3 (**C**). Total extracts of PAP7460 bacteria producing PulF from plasmid pCHAP7802 or its derivatives were treated with CuCl_2_ (see Materials and Methods) and analyzed by SDS-PAGE and immunoblot with anti-PulF_N_ antibodies. Migration of PulF, dimer (PulF_2_), the cross-reacting SlyD band, and Mw markers (M) (in kDa) is indicated. (**D**) Side view of the PulF monomer model highlighting the most reactive residues shown as blue spheres and non-reactive residues as red spheres. (**E**) Top view of the PulF dimer and trimer (**F**) models zooming on the interface and showing the residues that, when replaced by Cys, yield PulF dimers (in blue) or show the absence of dimers (in red). Individual monomers are shown in green, magenta, and cyan.

The AF2-multimer models and evolutionary contacts both predict involvement of TMS1 and TMS3 in PulF oligomerization. To test this prediction experimentally, we employed cysteine scanning mutagenesis and crosslinking. Bacteria producing PulF or its Cys-substituted variants (carried on plasmid pCHAP7802 and derivatives, Table S1) were treated with CuCl_2_ as an oxidizing agent (see Materials and Methods) and analyzed by SDS-PAGE and Western blot with anti-PulF_N_ antibodies (Fig. 5A through C). Although PulF contains two Cys residues at positions 123 and 129 of Cyto1 domain, no cross-linked species were observed for PulF^WT^ under oxidizing conditions (Fig. 5, WT). Several Cys substitutions in TMS1 yielded covalently cross-linked dimers upon oxidation, with the strongest signals observed for variants M166C and Y168C (Fig. 5A). Weak dimerization was observed for Cys residues in positions 219 and 223–225 in TMS2 (Fig. 5B). Strikingly, multiple Cys substitutions in the TMS3 led to dimer formation, with the most intense signals observed for variants E370C and L393C (Fig. 5C). When co-produced with the T2SS components, all of the Cys-substituted variants were fully functional for pullulanase secretion (Fig. S6A) and promoted assembly of PulG pili on the bacterial surface (Fig. S6B). A notable exception was variant PulF L372C, which showed the absence—degradation—of PulA and PulG (Fig. S6B, red label).

We examined the localization of residues that showed strong dimerization signals (in blue) or no dimerization (in red) on the models of PulF monomer (Fig. 5D), dimer (Fig. 5E), and trimer (Fig. 5F). Remarkably, the most reactive Cys residues at positions 168 in TMS1 and 370 in TMS3 are facing each other at the dimer and trimer interface, in agreement with the models (Fig. 5, blue labels). Conversely, a clear absence of crosslinking for PulF variants with Cys at positions 364, 375, 380, or 382 of TMS3 (Fig. 5, red labels) was consistent with orientation away from the predicted dimer and trimer interfaces. Since these data alone did not allow us to distinguish between dimer and trimer models, we tested additional contacts predicted by the highest scoring trimer models.

### Position-specific Cys cross-linking validates the PulF trimer model

**Fig 6:**
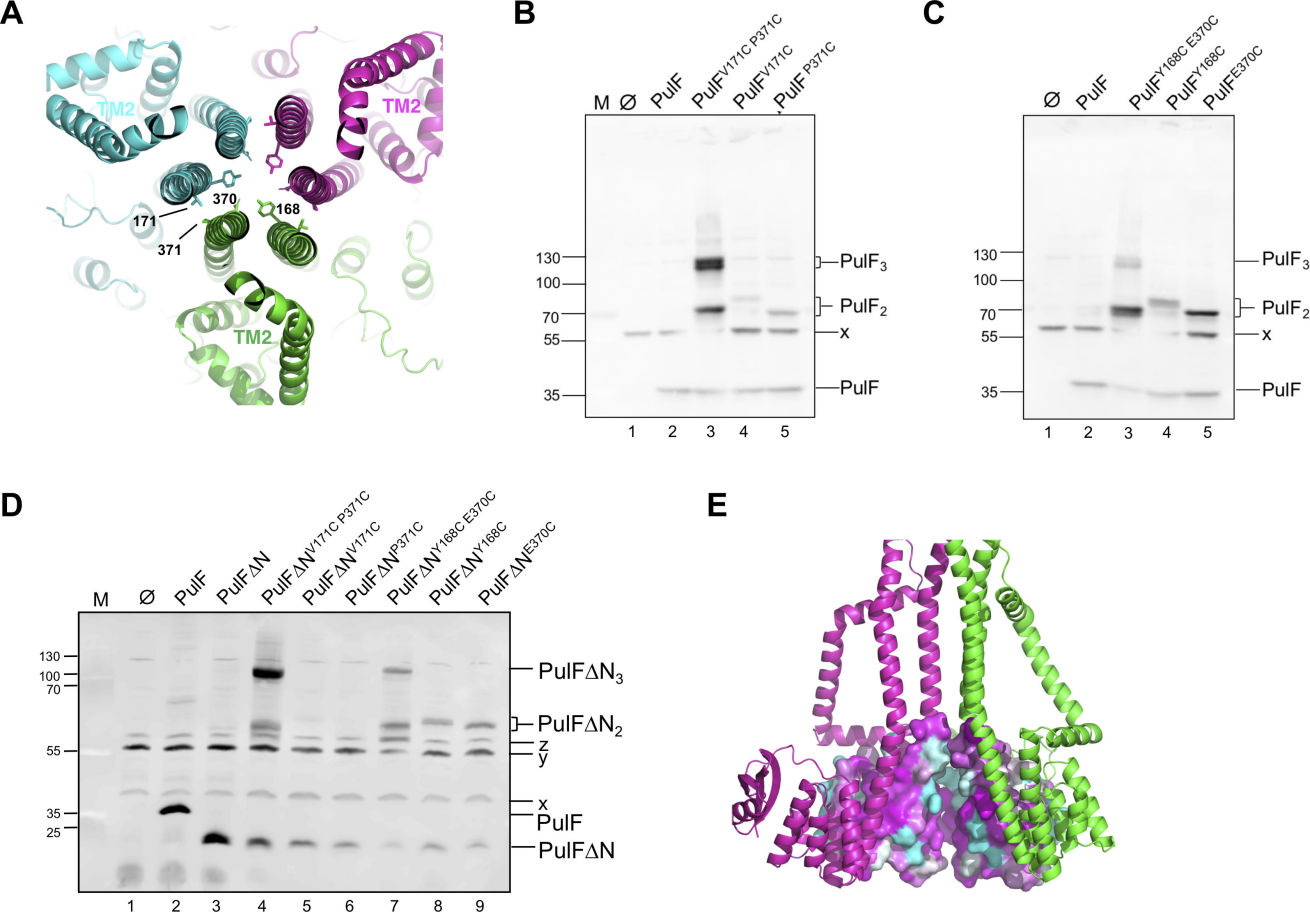
The PulF trimer model and validation. (**A**) Top view of the PulF trimer model showing the Cys substituted residues at the interfaces of PulF monomers shown in green, magenta, and cyan. (**B, C and D**). Total extracts of CuCl_2_-treated *E. coli* co-producing the T2SS lacking *pulF* (pCHAP8252) and PulF^WT^ (**B, C**) or PulFΔN (**D**) or their Cys variants encoded on pCHAP8259 or derivatives, analyzed on 4%–15% SDS gradient gels and Western blot with anti-PulFc antibodies. x, y, z, anti-PulFc cross-reacting bands. (E) Side view of the trimer with space-filling representation of Cyto1 and Cyto2 showing that conserved patches are buried. For clarity, the third protomer is not displayed.

A remarkable feature of the trimer configuration is the central channel, delineated by TMS1 and TMS3, with polar residues Y168 (TMS1) and E370 (TMS3) pointing toward its lumen (Fig. 6A). The trimer model predicts the proximity (4 Å distance) of specific residues at the interface between protomers (P), notably V171 in TMS1 of P and P371 in TMS3 of P + 1 (Fig. 6A). To test this prediction and to covalently stabilize the putative oligomer, we introduced Cys substitutions in these positions and produced single and double Cys substituted PulF variants in the presence of the remaining T2SS components. The bacteria were treated with CuCl_2_ (see Materials and Methods), and their total extracts analyzed on a denaturing 4%–15% polyacrylamide gradient gel (Fig. 6B and C). Single-substituted PulF^V171C^ and PulF^P371C^ remained mostly monomeric, as expected (Fig. 6B, lanes 4,5) although PulF^P371C^ also formed some dimers. This residue is adjacent to E370 and is located in TMS3, which showed high overall reactivity in Cys crosslinking studies (Fig. 5). Considering the theoretical Mw of the PulF dimer of 88,372 Da, these dimeric species, like the monomeric PulF, migrated proportionally faster with apparent Mw around 75 kDa. Oxidation of the double Cys variant PulF^V171C-P371C^ stabilized the trimer as the most abundant form, as well as some dimeric forms (Fig. 6C, lane 3), presumably products of incomplete oxidation, consistent with the model. The trimeric species also migrated faster than their predicted Mw of 132,558 Da, proportionally to the migration of the PulF dimer and monomer (~115 kDa). The PulF^V171C-P371C^ trimer formed a doublet on SDS-PAGE, the two bands possibly corresponding to the fully closed trimer form (A-B-C-A) and to an open form (A-B-C) resulting from incomplete oxidation. No higher Mw oligomers were detected, arguing against the tetramer model, which also predicts lateral contacts between V171 and P371 (Fig. S7). Moreover, in the tetramer model, the large distance between luminal Y168 and E370 residues would be incompatible with the observed dimer and trimer cross-linking (Fig. S7B).

We next analyzed the Cys variants Y168C in TMS1 and E370C in TMS3, which generated the most abundant dimers in single Cys crosslinking experiments. Combining these Cys substitutions in variant PulF^Y168C-E370C^ yielded two dimeric species, which migrated similarly to the dimers formed by the single Cys variants PulF^Y168C^ and PulF^E370C^ (Fig. 6C, lanes 3–5). The PulF^Y168C-E370C^ variant also formed two trimeric forms (Fig. 6C, lane 3), consistent with the proximity of Y168 and E370 pointing toward the lumen of a central channel. However, trimers were less abundant than dimers (Fig. 6C, lane 3), possibly due to the competition between intra- and inter-protomer crosslinking, or to altered conformational states less favorable for trimer crosslinking.

The Cys crosslinking analysis of the PulFΔN variant indicated a preserved trimeric structure of the PulF core in the absence of the N-domain (Fig. 6D). The variant PulFΔN^V171C-P371C^ formed abundant trimers and lower levels of covalent dimers (Fig. 6D, lane 4) when only monomeric species were observed with the single-substituted PulFΔN^V171C^ and PulFΔN^P371C^ (Fig. 6D, lanes 5 and 6). These results confirm that the peripherally localized N-domain is not required for PulF oligomerization. PulFΔN^Y168C-E370C^ yielded covalent dimers and trimers (Fig. 6D, lane 7), while oxidation of PulFΔN^Y168C^ and PulFΔN^E370C^ produced dimers, like in the corresponding PulF variants. Like in PulF, the formation of PulFΔN^Y168C-E370C^ trimers was less efficient compared to PulFΔN^V171C-P371C^.

Collectively, these results strongly support the trimeric state of PulF. In addition, the trimer model displayed the most favorable arrangement of the subunits as illustrated in Fig. 6E showing the buried conformation of the conserved patches of the Cyto1 and Cyto2 domains.

### Molecular dynamics and functional analysis of PulF oligomers

**Fig 7:**
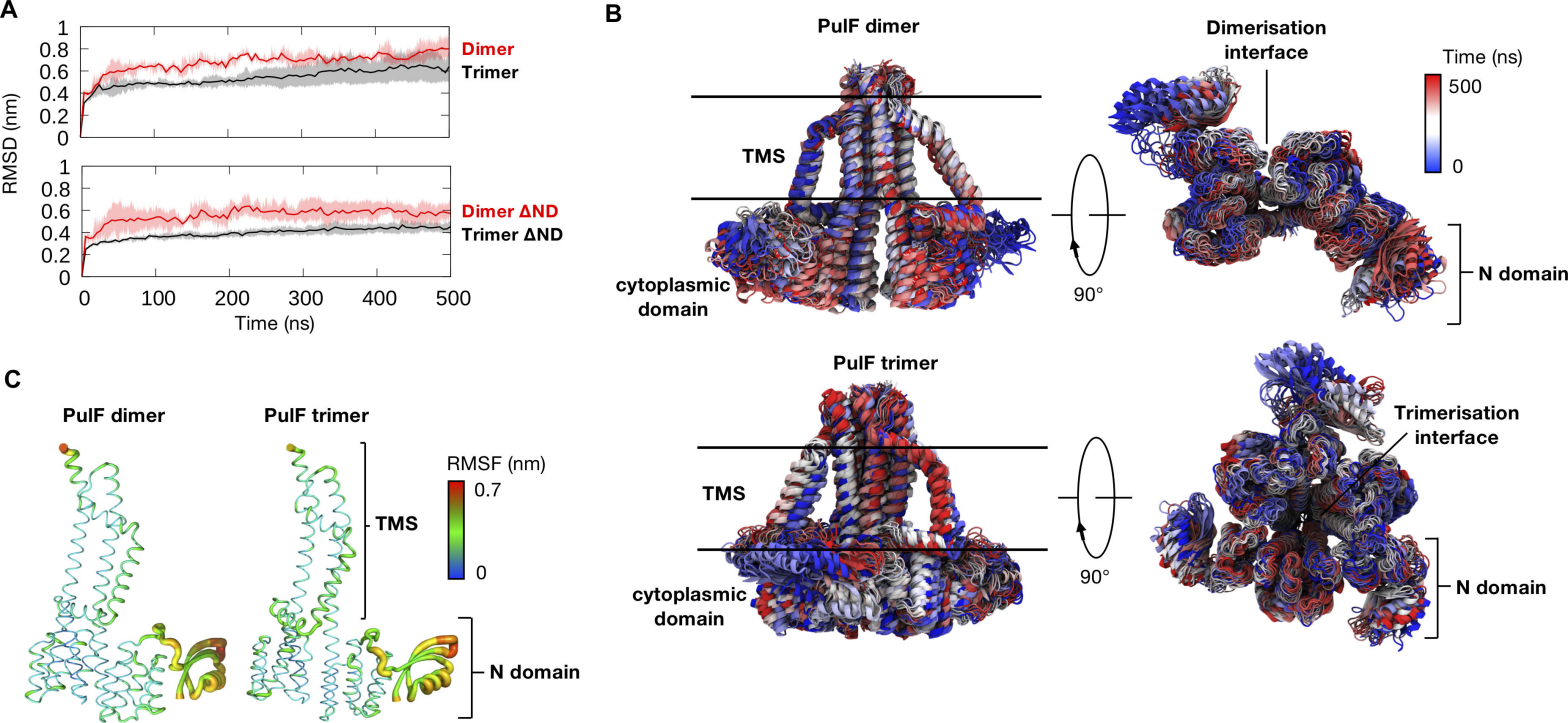
The dynamics of the PulF dimer and trimer from MD simulations. (**A**) PulF dimer and trimer models were inserted into an *E. coli* model membrane, and three independent 500 ns simulations were performed. Backbone RMSD of the dimer (red) and trimer (black) for full-length protein (top) or in the absence of the N-domain (bottom) along the MD trajectory. Average values are shown by the solid lines and the standard deviations between repeat simulations are shown as shaded regions. (**B**) Overlaid snapshots of the dimer (top) and trimer (bottom) every 10 ns showing the dynamics of the N-domain. The color represents the time progression during simulations from 0 ns (blue) to 500 ns (red). (**C**) Per-residue root mean square fluctuation (RMSF) values mapped onto the structure of PulF from simulations of dimer (left) and trimer (right).

To analyze PulF behavior in a native membrane environment, we embedded the PulF dimer and trimer models within a bilayer closely mimicking the lipid composition of the *Escherichia coli* inner membrane (47) and performed a series of all-atom MD simulations (see details in Materials and Methods). The trimer was more stable in its native membrane environment compared to the PulF dimer model, as indicated by the lower RMSD values with and without the N-domain (Fig. 7A), further supporting the trimer as the most probable oligomeric state of PulF. The most mobile PulF component during MD simulations was the N-domain which is connected to the core cytoplasmic and TM domains *via* a long flexible loop. The N-domain moved freely relative to the rigid core and the per-residue root mean square fluctuation (RMSF) values of the N-domain are significantly higher than for the rest of the protein (Fig. 7B and C). This is highlighted in the superimposed snapshots of the PulF dimer and trimer over the 500 ns time course of simulations. Interestingly, there was no significant difference in N-domain dynamics between dimer and trimer simulations (Fig. 7B and C). Increased mobility was also observed at the C-terminal helix facing the periplasm (Fig. 7C).

### PulF interactions with model membranes

**Fig 8:**
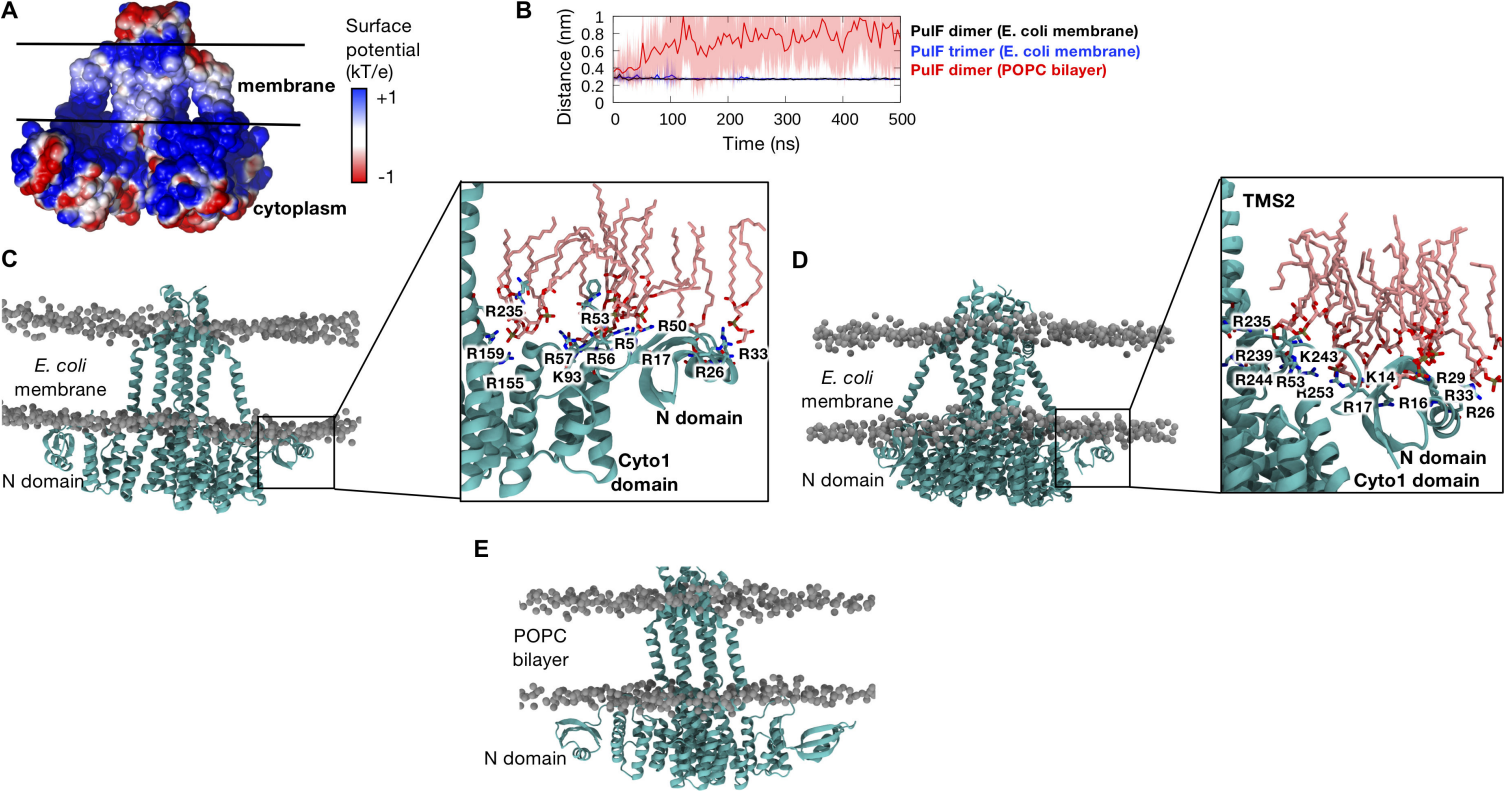
Interactions of PulF with the membrane. (**A**) Electrostatic surface potential of PulF dimer showing the high density of positive charges around the N-domain and the cytoplasmic domains facing the membrane. (**B**) Minimum distance between the N-domain (residue 1–45) and the phosphate groups on the lipid molecules throughout the simulations, comparing the PulF dimer (black) and trimer (red) in *E. coli* membrane with a control simulation of the PulF dimer in a POPC bilayer. Solid lines show average over two monomers and three independent repeats, and shaded areas indicate the standard deviation. (**C**) Central structure of the top cluster based on clustering analysis showing the PulF dimer in teal and lipid phosphate groups in gray for the simulations in *E. coli* model membrane. Enlarged image shows positively charged residues on the N-domain and Cyto1 domain that interact with negatively charged lipids (PVPG and CL) shown in pink CPK wireframe format. (**D**) Similar clustering and membrane interaction analyses performed on simulations of the PulF trimer. (**E**) Central structure of the top cluster based on clustering analysis for the control simulations in a POPC bilayer.

We next focused on how PulF interacts with membrane bilayers. PulF has a high density of positive charge on the N-domain and cytoplasmic domains facing the membrane, as demonstrated by the electrostatic surface potential (Fig. 8A), which may contribute to protein-lipid interactions. As such, we measured the minimum distance between the N-domain and the surface of the membrane and found that it remained lower than 4 Å throughout the entire simulations, indicating constant interactions, both in the PulF dimer (Fig. 8B, black line) and trimer (Fig. 8B, blue line). As a control, we performed a similar set of simulations of the PulF dimer embedded within a simple bilayer containing only 1-palmitoyl-2-oleoyl-phosphatidylcholine (POPC). Strikingly, the distance between the N-domain and the surface of the POPC bilayer consistently increased as the simulations progressed (Fig. 8B, red line), reflecting detachment of the N-domain from the membrane surface in the absence of anionic 1-palmitoyl-2-vaccenyl-phosphatidylglycerol (PVPG) and cardiolipin (CL). This is also reflected by an increase in mobility of the N-domain as depicted by the higher RMSF and RMSD values (Fig. S8). A slight loss in secondary structure was also observed in the POPC-only environment (Fig. S9), which could be caused by the lack of stabilizing interactions with the membrane. Snapshots from clustering analysis of dimer and trimer simulations (Fig. 8C and D) reveal specific contacts of multiple positively charged, mostly Arg, residues with PVPG and CL lipid head-groups in the *E. coli* membrane model. In contrast, the N-domain showed no binding to the neutral POPC membrane (Fig. 8E). Overall, our simulations reveal the importance of anionic lipids in stabilizing the PulF oligomer in its native membrane environment.

### Analysis of the PulF channel

**Fig 9:**
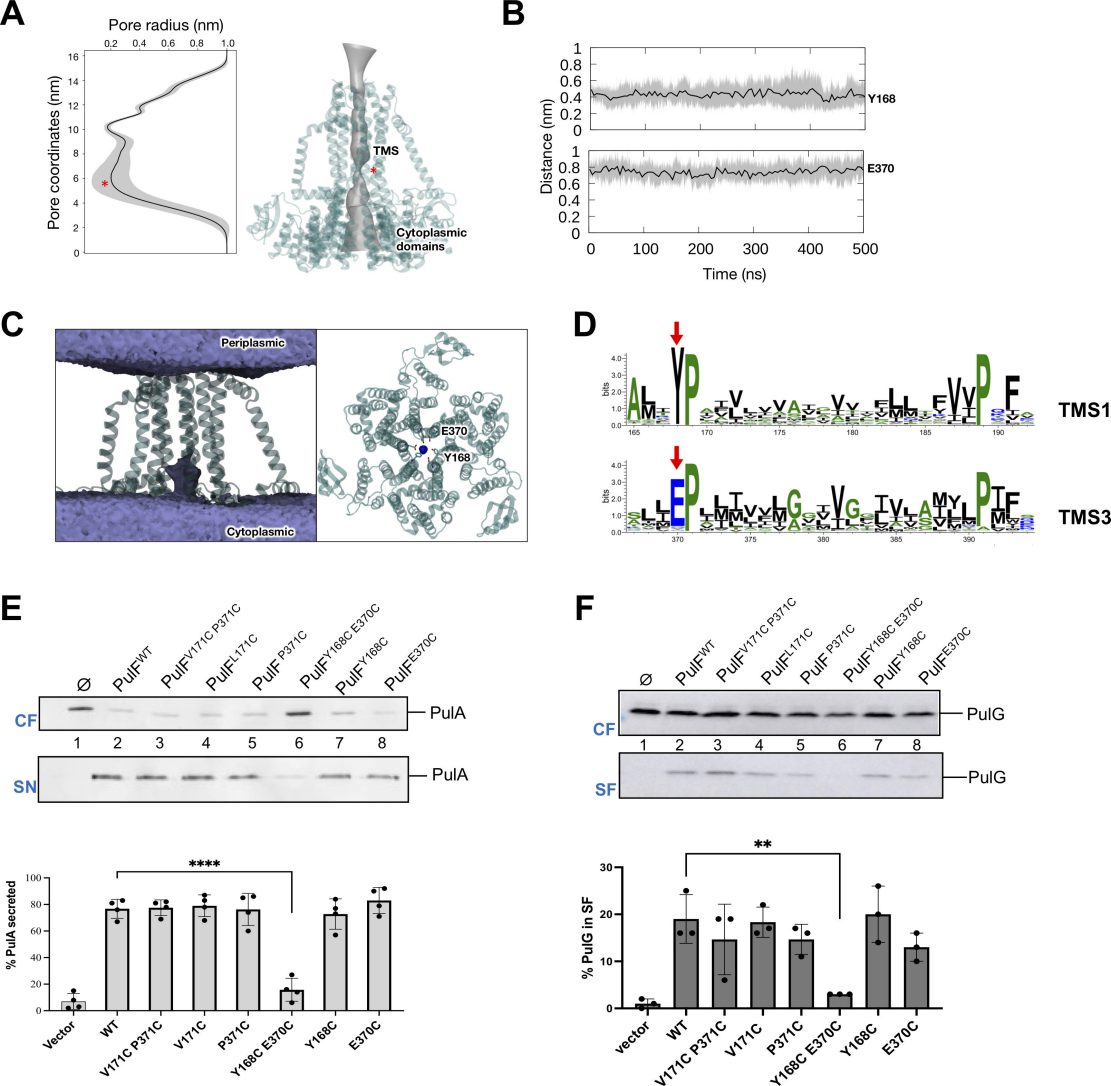
Properties and role of the PulF channel. (**A**) Average pore radius along the PulF channel throughout the simulations, with standard deviation shown as shaded regions. The position of the pore constriction is indicated by the red asterisk. The surface of the pore (gray) is projected onto the structure of the PulF trimer (teal). (**B**) Minimum distance between residues Y168 and E370 from the three protomers at the narrowest constriction of the pore during simulations. (C) The average volumetric map of water (purple, left, view parallel to the membrane) and Na^+^ ions (blue, right, view parallel to the pore) from simulations of the PulF trimer. (**D**) Gremlin analysis of GspF/PilC superfamily Weblogo showing the residue conservation in TM segments 1 and 3. Red arrows indicate conserved residues corresponding to Y168 and E370 in PulF next to the invariable Pro residues. (**E**) Double Cys substitutions of channel residues affect PulA secretion. Top: PulA levels in the cell fractions (CF) and supernatants (SN) of strain PAP5207 with plasmid pCHAP6539 and pCHAP8259 encoding PulF^WT^ or PulF variants indicated above each lane. Bottom: Quantitative analysis of PulA secretion (*n* = 4). (**F**) Double Cys substitutions of channel residues affect PulG pilus assembly. Top: PulG levels in cell- (CF) and sheared fractions (SF) in strain carrying pCHAP8252 (*ΔpulF*) and pCHAP8259 encoding PulF and variants indicated above each lane. Bottom: Quantification of PulG fractions assembled into pili (*n* = 3). The fractions of surface-exposed PulA and PulG (dots) and mean values (bar graphs) were calculated from at least three independent experiments. Statistical analysis was performed using ordinary One-way Anova with multiple comparisons. Significant differences compared to wild type are indicated: **P = 0.0064; ****P < 0.0001.

Pore analysis revealed a central channel demarcated by the trimer interface with a large, relatively breathable opening on the cytoplasmic side (Fig. 9A). This opening leads to the narrowest constriction around residues Y168 and E370, followed by a slightly wider, more rigid channel toward the periplasmic side of the membrane (Fig. 9A). Both residues Y168 and E370 from adjacent protomers remained close to one another during the entire simulations (Fig. 9B), maintaining the narrow constriction in the middle of the channel. Analysis of water and ion volumetric maps shows that water molecules are not able to permeate the channel due to the narrow constriction, while a sodium ion acts as a bridge between residues Y168 and E370 at the pore constriction (Fig. 9C). In addition to Na^+^ ions, the channel size would also be compatible with the passage of protons. Importantly, the polar residues lining the channel are highly conserved in the T2SS GspF and T4P PilC protein family, as shown in the Gremlin Weblogo of TMS1 and TMS3 (Fig. 9D). The absolute conservation of adjacent Pro residues suggests their crucial role in maintaining the conformation of the respective helices to define the channel constriction.

Despite high conservation, the single substitutions Y168C or E370C did not affect PulF function in our end-point essays. However, their combination in variant PulF^Y168C-E370C^ dramatically reduced PulA secretion and PulG pilus assembly (Fig. 9E and F). The defect was specific for channel residues since variant PulF^V171C-P371C^ was fully functional. Although no oxidizing agents were used in these functional assays, the analysis of cell fractions revealed that dimers formed spontaneously for variant PulF^Y168C-E370C^ (Fig. S10) suggesting that these residues are at least partially exposed to the oxidative periplasmic environment. These disulfide bonds might obstruct the PulF channel and thereby block proton or ion transport that might be required for endopilus assembly and protein secretion in T2SS. Alternatively, they might prevent conformational changes of the PulF trimer that are essential for its dynamic function.

### Model of PulF trimer in complex with PulE ATPase hexamer

**Fig 10:**
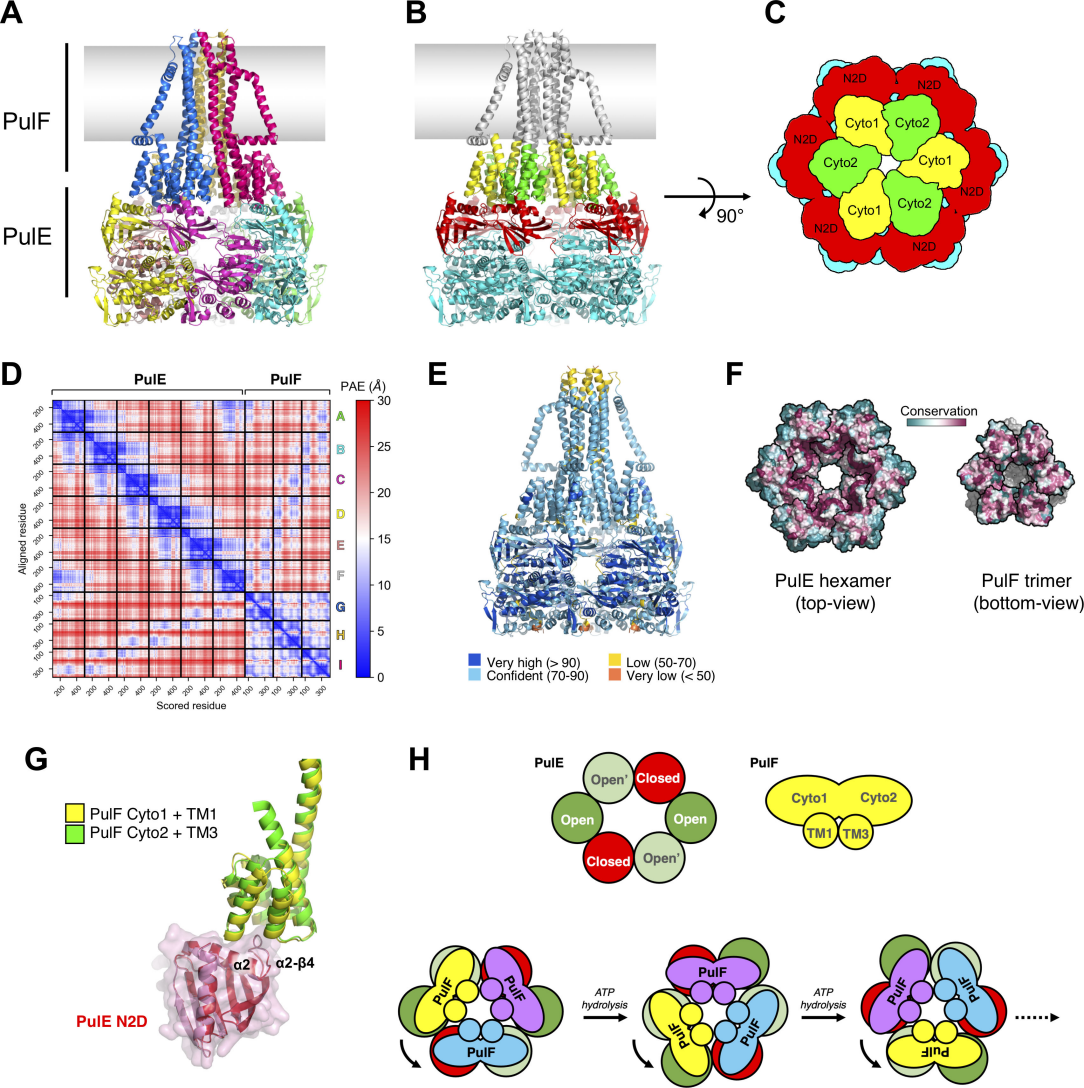
Model of PulF trimer in complex with PulE hexamer. (**A**) Side view of the AlphaFold2 PulE:PulF complex model with 6:3 stoichiometry. Each chain in the complex is colored differently. The approximate position of the IM is represented with a gray gradient. (**B**) Same complex as in (**A**) but with PulF domains colored in yellow (Cyto1) and green (Cyto2). PulE N2 domains (N2D) are colored in red and the C-terminal domains (CTD) in cyan. (**C**) Schematic view of the PulF domains Cyto 1 (yellow) and Cyto 2 (green), each in complex with a PulE N2D (red). The CTDs of PulE are in cyan. The view is seen from the membrane plane (i.e., rotated 90° compared to B). (**D**) Predicted Aligned Error (PAE) for the AF2 PulE_6_:PulF_3_ complex model. The first six (**A through F**) and last three (**G through I**) chains correspond to the PulE hexamer and the PulF trimer, respectively. Low PAE values are observed between PulE chains, between PulF chains, and also between PulE and PulF chains. (**E**) AF2 PulE_6_:PulF_3_ complex model colored by plDDT values. Apart from the tip of the PulF TM helices and the last C-terminal residues of PulE, the overall plDDT scores represent a confident prediction for the whole complex. (**F**) Surface representation of the PulE hexamer model (top view with N2Ds in front, left panel) and the PulF trimer model (bottom view with Cyto domains in front, right panel) colored by residue conservation, from highly conserved (dark magenta) to the least conserved (dark teal). For clarity, PulF TM segments are shown in gray. Each subunit is outlined in black. Highly conserved regions (magenta) are solvent exposed when each partner is considered separately but are part of the PulE:PulF interface in the model of the complex shown in (**A and B**). (**G**) Superimposition of the PulE:PulF interfaces in the AF2 model. The Cyto1 (yellow) and Cyto2 (green) domains of PulF interact in an identical manner with neighboring N2D of PulE. (H) Model for the rotation of PulF correlated with the ATP hydrolysis cycle of PulE ATPase. Each PulF is bound to two PulE subunits *via* their N2D. Upon ATP hydrolysis, the conformational change from the closed to the open state of two opposite PulE subunits in the hexamer (rotation ~60° of the N2D) would cause the simultaneous rotation of the PulF trimer.

To understand how the trimeric PulF may cooperate with the hexameric ATPase PulE, we used AF2-multimer to generate models of PulF in complex with PulE. As suggested by the hexameric GspE and related T4P PilB/PilT structures (34, 48), we modeled a PulE^ΔN1D^:PulF^ΔN^ complex with 6:3 stoichiometry (Fig. 10A and B). In the high-confidence AF2-multimer model, each Cyto domain of the PulF trimer interacts with the second N-terminal domain (N2D) of PulE (Fig. 10C). The AF2 model displays high confidence (ipTM of 0.67) with low PAE for all inter-subunit interfaces, i.e., PulE:PulE, PulF:PulF, and PulE:PulF (Fig. 10D and E). In the predicted complex, the interface regions of PulE and PulF display high levels of conservation (Fig. 10F) with a large buried surface area (~950 Å^2^ per PulF subunit) involving the α2 helix and the a2-β4 loop of PulE N2D; the two PulF cytoplasmic domains adopt the same interface with PulE N2D (Fig. 10G). In this PulE_6_:PulF_3_ model, the PulF trimeric structure is very close to the one predicted in the absence of PulE (Fig. S11A). When predicting an E:F complex with 6:2 stoichiometry, the resulting PulE:PulF topology is nearly identical to the one found in the 6:3 predicted model, but the PulF dimer displays a different structure than the one in the absence of PulE (Fig. S11B and C), rather similar to a PulF trimer but missing a third subunit and leaving two free PulE N2Ds.

In all PulE_6_:PulF_3_ models predicted by AF2, the PulE hexamer consistently exhibits a quasi C6-symmetry (average RMSD after circular permutation 0.35 Å). The structure of the related GspE from the *V. cholerae* T2SS was solved in two distinct hexameric configurations: with C6 symmetry or with C2 symmetry where opposite subunit pairs are in different conformations (Open, Open’, and Closed) (48). Interestingly, PulE hexamers predicted by AF2-multimer display a wider central opening when modeled in the presence of the PulF trimer compared to the one obtained for PulE alone (with similar ipTM ~0.69) and which superimposes well with the *Vc*GspE C6-symmetric structure (Fig. S12A and B). When compared to the C2-symmetric *Vc*GspE structure, the PulE hexamer in the best AF2 model for PulE_6_:PulF_3_ shows some similarity with the open conformation of GspE subunits where the N2D is slightly rotated and translated upward with regard to the CTD of the same subunit (Fig. S12C,D and S13B,C). When superimposing PulE subunits from the five PulE_6_:F_3_ models returned by AF2, it seems that a continuum of N2D orientations is explored from the closed (as observed in C6-symmetric *Vc*GspE) to the open conformation (Fig. S13D).

## DISCUSSION

GspF/PilC proteins are essential and among the least characterized components of T4F assembly systems, crucially positioned between the ATPase and pilin substrates. However, efforts to determine their structure met with limited success. In a remarkable effort to purify the complete T2SS complex of *K. pneumoniae*, PulF and pilins were not co-purified with the rest of the machinery (49), probably reflecting detergent-sensitive and dynamic interfaces. Here, we present a detailed structural model of this prototypical member of the fiber assembly platform protein family. The trimeric state of PulF, extensively validated through mutational and functional analyses, has important implications for the organization and molecular function of the assembly platform. Previous models based on cryo-ET and X-ray crystallography data considered a dimeric GspF complex (16) which was proposed to occupy the central position in the asymmetric cavity formed by the GspE ATPase hexamer (34). However, in these models, only a part of the protein was considered, comprising two cytoplasmic domains, but lacking the N-domain.

Here, the converging structural models provide the complete view of PulF including the N-domain with a ββαβ fold. We show that the N-domain is dispensable for function, nevertheless required for fully efficient PulG pilus assembly and PulA secretion. The non-essential role of the N-domain has also been observed in the T4P homologs PilC of *P. aeruginosa* (15) and PilG in *N. meningitidis* (50). A piliation defect of a *pilG* mutant mapping in the N-domain is suppressed by the removal of the retraction ATPase PilT in contrast to mutations mapping in the PilG core domain that completely inactivate the protein (14). The N-domain is the most mobile region that significantly contributes to overall PulF dynamics in our MD simulations. The flexibility of the β3-α2 loop connecting the N-domain to the PulF core allows for large N-domain movements and explains the reported difficulties in crystallization of full-length PulF homologs (21). *In vivo*, the β3-α2 loop appears to be protected from proteolysis, presumably due to its binding to the *E. coli* membrane surface *via* anionic phospholipids including CL, as revealed by *in silico* studies.

To understand the mechanistic role of the N-domain, we searched for its structural homologs in the Protein Data Bank (Fig. S14). Two of them are small proteins—GP2 from phage T7 (51) and YkzG from *Bacillus stearothermophilus* (52)—which bind to RNA polymerase *via* a conserved Arg residue exposed on the surface of its β-sheet. In GP2, this residue is required to inhibit transcript elongation, but it is absent in the PulF N-domain. It, thus, appears that structural homology does not inform further on the origin and/or function of this short domain of PulF. Coevolution analysis shows no evidence of N-domain interactions with other PulF regions, and biochemical data show comparable stability and oligomerization of PulF and the PulFΔN variant. To understand how the N-domain regulates or contributes to PulF activity, it will be important to identify its interacting partners among T2SS components.

Structural homologs of the PulF Cyto1 domain in the PDB include other members of the GspF/PilC family—PilC from *T. thermophilus* (RMSD 1.2 Å) (26) and TcpE from *V. cholerae* (RMSD 2.2 Å) (27), both essential for the T4P assembly (Fig. S15A). Interestingly, despite very low sequence identity (13%), structural similarity is also found with the soluble domain of SpoIIIAB (RMSD 2.8 Å), an essential sporulation channel protein in *Bacillus subtilis* that forms a secretion-like machinery with protein homologs from other bacterial secretion systems (Type II, III, and IV) (53). While monomeric in solution, this SpoIIIAB domain crystallizes as a tetramer with two distinct dimer interfaces but with a head-to-tail orientation of the monomers unlike the parallel orientation seen in EpsF or PilC dimer. Surprising structural similarity is also found between PulF Cyto1 and the C subunit of the *T. thermophilus* V-ATPase (53–55). The C subunit is a 3-domain protein organized as a pseudo-symmetric trimer (RMSD of ~2 Å between domains), where each domain exhibits a 6-helix bundle fold similar to the PulF Cyto1 (RMSD 3.4 Å over 90 aligned residues of the D2 domain, Fig. S15B). In V-ATPase, the C subunit holds a central position in the rotating axis between the catalytic V1 domain and the membrane-embedded L-12 ring of the V0 domain and was proposed to act as a socket and adaptor for reversible binding of the soluble ATPase V1 domain and the V0 membrane domain (54). Unlike the *T. thermophilus* C subunit, the socket formed by PulF Cyto1 and Cyto2 domains is permanently membrane-anchored *via* its TM segments. In addition to the structural similarities of GspE/PilB and the V1 domain of the V-ATPase (33, 56), the GspF/PilC resemblance to the C-subunit and the V0 domain suggests evolutionary and functional links between T4F assembly systems and the energy converting nanomachines.

The widely proposed dimeric state of PulF homologs (15, 29) has been incorporated into the cryo-ET models of T4P and T2SS (16, 57). Here, we used AF2 and AF2-multimer to model several possible PulF oligomeric states. We combined residue coevolution and conservation analysis to infer oligomerization interfaces. This analysis identified conserved patches on Cyto1 and Cyto2 that may be involved in oligomerization, while AF2 identified TMS1 and TMS3 as the main dimer interface. The model of the PulF trimer was fully consistent with residue conservation and position-specific Cys crosslinking, which proved as a powerful tool to validate structural models of the membrane-embedded PulF complex in an unbiased and comprehensive manner. Covalent capture of contacts at the protomer interface between positions 171 and 371 allowed us to validate the trimer and discard the dimer and tetramer models. The MEMSAT_SVM prediction of TMS3 as a pore-lining helix based on its sequence analysis (58) is also consistent with the channel-forming PulF trimer, while MD simulations additionally support the superior stability of the trimer compared to the dimer. Crosslinking of variants Y168C and E370C also supports the trimer model showing the proximity of these residues in the dimer and trimer interfaces. Their intra-protomer crosslinking may reduce the probability of inter-protomer contacts, thus explaining the higher dimer/trimer ratio for PulF^Y168C-E370C^ variant. Alternatively, this might indicate trimer asymmetry and conformational changes altering the distance between Y168 and E370 side chains, as supported by the final snapshots of three independent MD simulations of the PulF trimer (Fig. S16).

Despite an excellent fit of the AF2 trimer model with biochemical data, some Cys crosslinking results do not correlate with the predicted residue proximity. PulF variants with Cys substitutions mapping on the periplasmic end of TMS1 and TMS3 (e.g., PulF^V187C^ or PulF^L393C^) were highly reactive in the cross-linking assay. This is likely due to their permanent exposure to the oxidative periplasmic environment and to TMS3 flexibility in this region, as revealed by MD simulations (Fig. 7). Crosslinking of several PulF variants including M166C (TMS1), L372C, or L373C (TMS3) could reflect some flexibility and relative rotations of these TM segments. More puzzling is the PulF dimerization *via* L224C or A226C in TMS2, facing outward in the oligomer. Bringing these residues closer would require a dramatic conformational change of TMS2 swinging the entire D-arm toward the neighboring TMS2, which could occur *via* the flexible regions flanking the α8 and α9 helices on the periplasmic and cytoplasmic sides. Such a change might be induced by interactions with the pilin substrates or other partners in the system. However, the weak PulF dimerization *via* TMS2 (Fig. 5B) was observed in the absence of T2SS components, a context in which TMS2 contacts might involve interactions of PulF monomers in the membrane prior to assembly, or distinct PulF trimers.

Previous EM studies have led to proposals that the *N. meningitidis* T4P assembly platform protein PilG forms a tetramer (29). Although our data argue against the tetramer model, single particle analysis of purified PilG oligomers produced a low-resolution EM map with features highly reminiscent of our PulF trimer model. These include a conical shape with contours of the D-shaped arms defining lateral cavities, and the peripheral gold particles colocalizing with the N-domains (29).

In terms of symmetry, the PulF trimeric state is compatible with the hexameric ATPase PulE and its presumed 1:1 stoichiometry with PulL, whose cytoplasmic domain forms a stable complex with the PulE first N-domain (N1D) and serves as its membrane anchor (59). Multiple structures of the T4F ATPases reveal conformational changes of the central channel, which rotates clockwise in the case of assembly of the ATPase and counterclockwise in the disassembly of the ATPase PilT (34). A model generated based on the PilC Cyto1 domain proposes that the PilC dimer might fit into the two sub-pores in the center of the ATPase hexamer. Cycles of ATP binding and hydrolysis would induce rotation of these sub-pores, while generating an upward thrust and rotation of PilC (34). We generated models of trimeric PulF in association with hexameric PulE that revealed that each cytoplasmic domain of the PulF trimer bind to an individual N2 domain (N2D) of PulE with a conserved interface. Such a one-to-one pairing between Cyto domains of PulF and N2D of PulE would ideally ensure that any movement (rotation and/or uplifting) of a single N2D of the motor ATPase would be transmitted directly to PulF and, thus, to the assembly platform. In fact, in the PulE_6_:PulF_3_ models predicted by AF2-Multimer, the orientation of the PulE N2D displays some similarities with an open conformation of the ATPase subunit, previously described as a post-ATP hydrolysis stage in T4P PilB motor (60). Our model of the PulE:PulF complex is, thus, compatible with the previously described rotational model, where the rotation of the N2D of opposite PulE subunits upon ATP-hydrolysis would promote rotation of the three PulF subunits through the N2D:Cyto1/2 interfaces (Fig. 10H). Furthermore, the 3:6 association of PulF with the ATPase makes the trimer the common element of the stoichiometry puzzle in the T2SS machinery, from the hexameric ATPase at the IM to the pentadecameric secretin in the outer membrane (49, 61–63). By analogy, the same 6:3 stoichiometry is expected to exist in T4P for the PilB/T:PilC complex. However, T4P secretins are reported to mostly display C14 symmetry, while some alternate symmetry (C13/C15) has also been observed in some instances (64–66).

A similar model generated by AlphaFold-Multimer (43) was recently proposed for the PulF homolog ComGB from the *Streptococcus sanguinis* T4P (67). In this model, the ComGB trimerizes *via* a somewhat different interface, to form a wide hollow shaft, unlike the narrow central channel of the PulF trimer. Interestingly, ComGC lacks the N-domain but shares with GspF/PilC homologs the conserved residues in TMS1 (Tyr-Pro) and TMS3 (Gln-Pro instead of Glu-Pro). However, in the proposed ComGB trimer, the distance between these residues would be incompatible with cysteine crosslinking observed here. Additional studies are required to test this model and identify how platform proteins interact with pilins. Interestingly, our monomeric and trimeric PulF models would also be compatible with T4cP systems (such as the Tight adherence secretion system, Tad) where the two PulF paralogs, TadB and TadC (8, 68), each carrying a single Cyto domain, would assemble as a trimer of hetero-dimers, eventually forming a similar ring of six cytoplasmic domains and TMS.

MD simulations of PulF in the *E. coli* model membrane revealed that several regions of PulF strongly interact with anionic phospholipid headgroups. Such contacts are much weaker in a POPC membrane, leading to increased dynamics, particularly for the N-domain. Specific contacts of positively charged residues with PVPG and CL in the *E. coli* membrane lead to an enrichment of this lipid bound to PulF. The N-domain makes an important contribution to this enrichment, which might contribute to the functional defect of the PulFΔN variant. Another region involved in anionic lipid binding is the α9 helix lining the cytoplasmic face of the IM. In *P. aeruginosa* PilC, mutations in the α9 helix equivalent affect T4P function in twitching motility, but also the PilC levels (15), suggesting that membrane binding *via* CL plays a stabilizing role. Studies of *Vibrio* T2SS demonstrate that CL stimulates the EpsE ATPase activity (69). Given the intimate contacts of PulF with PulE, the CL bound to PulF might participate in this stimulation. CL binds to other ATPase-driven molecular motors, including the ATP synthase (70) and SecY translocon, stimulating the ATPase activity of SecA (71). CL is required for the proton motive force (PMF)-driven protein translocation and may act as a proton buffer or reservoir (71), contributing to the proton ratchet mechanism during protein export (72).

The most important feature of the trimeric PulF revealed by modeling, MD, and functional analysis is the central, water impermeable channel, constricted at the level of polar residues Y168 and E370, which are conserved in all GspF and PilC homologs in T2SS and T4aP. *In silico*, the PulF channel is predicted to accommodate protons or sodium. At least two previous studies demonstrated that protein secretion requires the PMF, as the ionophore CCCP blocks secretion in pulse-chase assays in a rapid and reversible manner (73, 74). Although, to our knowledge, the role of membrane potential in T4P assembly has not been tested in kinetic assays, PMF depletion reduces the speed of pilus retraction in *N. gonorrhoeae* (75) and treatment of *N. meningitidis* with drugs that affect the IM Na^+^ gradient inhibit T4P formation (76). The dramatic functional defects observed when a disulfide bridge is formed between PulF^Y168C^ and PulF^E370C^ may be due to an obstructed channel, arguing for its essential role. Alternatively, Cys crosslinking might block the trimer in a specific state and prevent conformational changes required for its function.

The GspE ATPase is considered as the motor of T2SS as ATP binding and hydrolysis promote its conformational changes essential for secretion (69). Our results suggest that the GspF channel might mediate the essential role of the ion gradient in T2SS (56). The requirement of an open PulF channel for PulG pilus assembly suggests that membrane potential also fuels the T4P assembly, possibly by driving a rotational movement as proposed earlier (77). The role of the PMF in T2SS finds parallels in other secretion systems. In T3SS, the InvA membrane component of the so-called export apparatus and FlhA in the flagellar system have been proposed to play the role of a proton or sodium channel lined with conserved polar residues (78, 79). Similarly, in the T9SS, polar residues important for function have been identified in the TM segments of the GldLM/PorLM complex (80) and MotA-MotB (81) or PomA/PomB (82) flagellar stators.

Elucidating the mechanism of pilus assembly and type II secretion requires detailed structural and biochemical studies of GspF and its interplay with GspE and other AP components. Our model of PulF and its complex with PulE offer a framework to understand how these components interact with the pilin substrates during the initiation and elongation steps of assembly. Modeling and MD simulations in model membranes will be instrumental to study the energetics and dynamics of protein, membrane, and solute components that probably all contribute to orchestrate the dynamic events in the active site of T4F assembly systems.

## MATERIALS AND METHODS

### Bacterial strains and media

Bacterial strain DH5αF’*lacI^Q^* was used for most cloning purposes. PulG pilus assembly and PulF oligomerization were studied in strain PAP7460 (20) and PulA secretion in strain PAP5207 (83). For the BACTH assay, we used the *cya* mutant strain DHT1 (84). Bacteria were cultured in LB media, liquid, or supplemented with 1.5% agar (85). Antibiotics were added, as required: ampicillin (Ap) or carbenicillin (100 μg.mL^−1^), chloramphenicol (Cm) (25 μg.mL^−1^), or kanamycin (Km) (50 μg.mL^−1^). Inducers d-Maltose (0.4%) (for *pul* promotors) or isopropyl-thio-β-d galactoside (IPTG) (1 mM) for *lacZ* promotor were added as required.

### Plasmid constructions

Plasmids used in this study are listed in Table S1. Plasmid pCHAP8259 contains the *pulF* gene, amplified from plasmid pCHAP231 carrying the *pul* locus of *K. oxytoca* (86) using the high-fidelity Q5 DNA polymerase (NEB) and placed under the control of *lacZ* promoter. To obtain plasmids p15A/Cm^R^ from pCHAP6454 to pCHAP6466 (except pCHAP6658), the corresponding ColE1/Ap^R^ plasmids were digested with *Eco*RI and *Xba*I, and each fragment carrying the *pulF* gene with a cysteine codon substitution was ligated with the *Eco*RI/*Xba*I digested pSU18 vector. For mutagenesis, Quick Change method was used typically with 18 PCR cycles (30 s at 96°C, 30 s at 50°C, and 3 min at 72°C). After *Dpn*I digestion, 10–20 μL of the reactions were introduced into ultra-competent DH5α F'*lacI^Q^* cells and transformants were selected on LB Cm or Ap plates. For the secretion assay, a derivative of pCHAP8185 with a complete deletion of the *pulF* gene named pCHAP6539 was constructed as follows. Two separate PCRs were performed to amplify the DNA region upstream and downstream of the *pulF* gene with primers PF173-PF178 and PF180-181 and digested with the *Eco*RI and *Hin*dIII, respectively. The two digested fragments were ligated into the *Eco*RI and *Hin*dIII-digested pUC18 vector. The *Bsu*36I-*Not*I fragment of the resulting plasmid pCHAP6541 replaced the pCHAP8185 *Bsu*36I-*Not*I B fragment to give pCHAP6539. The same strategy was used to delete the N-domain *pulF* region in plasmid pCHAP6601 (pCHAP8185 with *pulF*∆1–54). The restriction site *Hin*dIII in the *pulF* gene was used for the subcloning in the pUC18 vector to obtain the intermediary construct pCHAP6600. To obtain the plasmid encoding the N-terminally truncated PulF (PulF55-401, pCHAP6469), the PCR fragment was ligated in the vector after digestion with *Eco*RI and *Bam*HI. The *Eco*RI-*Bam*HI fragment of this plasmid was cloned in the digested ColE1/Ap^R^ vector to yield pCHAP6650. Table S2 lists the oligonucleotides (Eurofins Genomics) used in this study. All the resulting plasmids were purified using Nucleospin plasmid kit (Macherey-Nagel) or Qiagen and verified by DNA sequencing (GATC and Eurofins Genomics).

### SDS-PAGE and immunodetection

Proteins were analyzed by SDS-PAGE with Tris-glycine (87) or Tris-Tricin buffer systems (88). Proteins were transferred to a nitrocellulose membrane (ECL Amersham, Cytiva) and probed with primary antibodies. Two types of polyclonal anti-PulF antibodies were used: (i) PulF_N_ sera raised against His_6_-PulF^1-136^ (N-terminally tagged fragment of PulF comprising residues 1–136) and (ii) custom PulF_C_ antibodies raised against a mixture of three PulF peptides 55-MRRTSARDLALVTRQ-69, 102-GVRGKVLEGHSLAEAMR-118, and 295-SNAWAKRQLEAASDAVREGVS-315 (Proteogenix, France). Polyclonal anti-PulG (89) and anti-PulA (90) antibodies were described previously. Blots were probed with secondary goat anti-rabbit antibodies coupled with horse-radish peroxidase (Amersham) and developed by ECL2 kit (Thermo). The fluorescence signals were recorded on a Typhoon FLA9000 phosphor-imager (GE).

### PulG pilus assembly and PulA secretion assays

Piliation assays were performed in *E. coli* strain PAP7460 transformed with one of the following plasmids: pCHAP8185, pCHAP8252, or pCHAP6601 and either vector alone or pCHAP8259 derivatives carrying *pulF* were grown 48 h at 30°C on LB agar containing Ap, Cm, and 0.4% maltose. Bacteria were harvested in LB and normalized to OD_600nm_ of 1. Pili were sheared by a 1 min vortex treatment, and bacteria were pelleted for 5 min at 16,000 × *g*. Pellets were resuspended in SDS sample buffer at 10 OD_600nm_. mL^−1^, and the supernatant was cleared from the remaining bacteria in a second 10 min centrifugation. The cleared supernatant was transferred to a new Eppendorf tube and precipitated with 10% tri-chloro-acetic acid for 30 min on ice. Pellets were collected by 30 min centrifugation at 16,000 *× g*, washed twice with acetone, air-dried, and taken up in SDS sample buffer at a concentration of 10 OD_600nm_ equivalents per mL. Equivalent volumes of bacteria and pili fractions from 0.1 OD_600nm_ of bacteria were analyzed by SDS-PAGE and Western blot with anti-PulG antibodies. PulA secretion was analyzed in bacterial strain PAP5207 containing either pCHAP8185 or its *pulF* deletion derivatives pCHAP6539 or pCHAP6601 and pCHAP8259 or derivatives encoding PulF variants. Bacteria were cultured in LB supplemented with appropriate antibiotics, 0.1 V of M63 salts and 0.4% maltose to OD_600nm_ of ~2 and chilled on ice. Bacteria were normalized of OD_600nm_ of 1 and centrifuged 10 min at maximum speed in table-top Eppendorf centrifuge. Pellet fraction was resuspended in SDS sample buffer, and supernatant was transferred to a new tube and centrifuged for another 10 min. An aliquot of the supernatant was mixed with an equal volume of 2 × SDS sample buffer. Fractions corresponding to the same OD of bacteria were analyzed by SDS-PAGE and western blot with anti-PulA antibodies, developed with ECL2 and imaged using Typhoon FLA9000. The fluorescence signals were quantified with ImageJ. The percentage of proteins found in the extracellular fractions was calculated for each sample. Data were analyzed and plotted using the Prism GraphPad software.

### Cysteine cross-linking

Plasmids pCHAP7802 encoding wild-type PulF or its cysteine-substituted derivatives were transformed into the *E. coli* PAP7460 strain. Bacteria were grown overnight in LB in the presence of ampicillin at 100 µg mL^−1^ at 30°C. The next day, 200 µL of the overnight culture was inoculated into 5 mL of fresh media and incubated at 30°C to an OD_600nm_ of ~2. One OD_600nm_ of bacteria was harvested by centrifugation (12,000 × *g* for 5 min) and washed twice with phosphate buffer saline (PBS). Bacteria were resuspended in 1 mL of the cross-linking buffer (50 mM MOPS pH 7.0, 5 mM MgCl_2_, 10% glycerol) and incubated 10 min at 23°C in a thermomixer with shaking at 750 rpm. For oxidation, 30 μL of 10 mM CuCl_2_ was added to each sample. After an incubation of 23 min at 23°C, the reaction was stopped by adding 45 μL of 0.5 M EDTA. Bacteria were centrifuged and resuspended in 100 µL of the SDS sample buffer (87). Samples were boiled for 10 min and 20 μL was analyzed by 10% glycine, or 8% tricine SDS-PAGE, or using 4%–15% TGX stain-free protein gels (Biorad). Proteins were transferred on nitrocellulose membranes and probed with anti-PulF_N_ or anti-PulF_C_ antibodies. Each variant was checked for cross-linking three times, and the variants which showed a reproducible cross-linked band were considered as positive.

### Bacterial two-hybrid analysis

For the bacterial two-hybrid analysis (46), bacteria of strain DHT1 were co-transformed with plasmids pUT18C and pKT25 and/or their derivatives (Table S1). Bacteria were grown for 48 h at 30°C on LB plates containing Ap (100 μg mL^−1^) and Km (50 μg mL^−1^). For each strain, 1 mL of LB containing Ap and Km was inoculated with randomly picked colonies and cultured at 30°C for 7 h. In the evening, 0.015 mL of the precultures was inoculated into 5 mL of LB containing Km (50 μg mL^−1^), Ap (100 μg mL^−1^), and 1 mM IPTG. Bacteria were cultured to stationary phase (OD_600nm_ of ~2), chilled on ice, and β-galactosidase activity was measured as described (85). Briefly, after measuring the *A*_600nm_ of the cultures, defined culture volumes (V) were diluted into the Z buffer containing M63 salts, 0.0037% SDS and 10 µM of DTT to a final volume of 1 mL. These 1 mL reaction mixtures were supplemented with 20 μL of CHCl_3_, and vortex treated for 10 sec to permeabilize bacteria. The reaction mixtures were prewarmed at 28°C for 10 min and 0.2 mL of o-nitrophenyl-β-d-galactoside (ONPG) (0.4%) solution in Z buffer was added to start the reactions. When the yellow color developed, the reactions were stopped by adding 0.5 mL of 1 M Na_2_CO_3_ solution and the reaction time (T) was recorded. The absorbance of the reaction mixtures was measured at 420 and 550 nm. Two independent measures of β-galactosidase activity were performed for each colony. The β-galactosidase activity was calculated in Miller units according to the formula: [*A*_420nm_ – (1.75 × *A*_550nm_)] × 1,000/V (mL) × *T* (min) × *A*_600nm_. To assess the Lac phenotype on plates, 5 µL of the overnight cultures was spotted on LB plates containing the antibiotics, X-Gal and IPTG. After 24 h of incubation at 30°C, the plates were stored at 4°C until the images were taken.

### Analysis of residue co-evolution and secondary structure prediction

Long-range contact predictions from residue co-evolution were obtained with the Gremlin server (http://gremlin.bakerlab.org/) (91). Using the *Klebsiella* PulF sequence as query, a Multiple Sequence Alignment (MSA) of 2553 homologous sequences was generated which corresponds to 4.75 effective sequences per position. From this MSA, Gremlin predicted 195 inter-residue contacts (|*i−j*| > 3) with a Scaled score ≥1. Secondary structure predictions of *Klebsiella* PulF were obtained by the PsiPred server (35).

### Modeling of the PulF N-domain

The PulF_1-57_ sequence was submitted to the Robetta server (http://robetta.bakerlab.org/) for structure prediction using the RosettaCM option (36). The first 3D model returned by Robetta was considered the best solution and used henceforth. Separately, an *ab initio* model was also built from Gremlin predicted contacts using the ARIA-EC approach (https://gitlab.pasteur.fr/bis-aria/ariaec). In short, inter-residue contacts are first converted into distance restraints and supplemented with restraints imposing secondary structures, derived from PSIPRED predictions (35). Then, restraints are supplied to ARIA (92) for iterative cycles of structure calculation and restraints analysis. The resulting ensemble of models is submitted for a final refinement in water (93). Of note, all contact predictions from Gremlin involving PulF_1-57_ were used for modeling, no probability cutoff of score was applied for selection contacts used as restraints. See reference (94) for details on the ARIA-EC approach applied to *de novo* modeling. Structural homologs of the PulF N-domain in the PDB were found using the DALI (95) and COFACTOR (96) servers.

### Homology modeling of PulF Cyto1 and Cyto2 domains

We used Modeller (97) to build atomic models of PulF_57-165_ and PulF_260-367_ using the crystal structure of *V. cholerae* EpsF_62-170_ (PDB id 3C1Q) as a structural template (21). For comparison with Gremlin contacts prediction, 3D models were also built for PulF Cyto1 and Cyto2 homo-/hetero-dimers based on the dimeric structures of either *Vc* EpsF_62-170_ (PDB id 3C1Q) or *Tt* PilC_61-162_ (PDB id 2WHN) (26).

### Modeling of full-length PulF monomer and oligomers with AlphaFold2

Models of PulF monomeric and oligomeric complexes were predicted with AlphaFold version 2.3 (38) using the ColabFold (98) implementation. In short, using the PulF sequence as query, ~10 k homologous sequences were retrieved and aligned using MMseqs2 (99). For the modeling of oligomers, the resulting multiple sequence alignment (MSA) was copied 2, 3, or 4 times with padding to create a single MSA for the dimer, trimer, or tetramer, respectively. Three-dimensional atomic models were generated using AF2.3 using the “*ptm*” model parameters for monomeric PulF and the “*multimer*” parameters (v2.3) for oligomers and further relaxed with OpenMM (100). The best 3D models were chosen as the ones with the best pTM for monomeric PulF or best multimer score (0.2*pTM + 0.8*ipTM) for oligomers, which are the recommended confidence metrics returned by AF2. Interface analysis of PulF oligomeric models was performed with the PISA server (101).

### All-atom molecular dynamics simulations

The PulF dimer structure generated by AF2 (38) was simulated in an IM model of *E. coli* containing 70% 1-palmitoyl-2-oleoyl-phosphatidylethanolamine (POPE), 20% 1-palmitoyl-2-vaccenyl-phosphatidylglycerol (PVPG), and 5% 1-palmitoyl-2-vaccenyl-3-palmitoyl-4-vaccenyl-diphosphatidylglycerol also known as cardiolipin (CL) (102–105). As a control, the protein was also simulated in a pure 1-palmitoyl-2-oleoyl-phosphatidylcholine (POPC) bilayer. The protein and membrane were assembled using the CHARMM-GUI Membrane Builder (106). The CHARMM36m force field was used to parametrize the PulF model (107). The system was solvated with TIP3P water molecules and neutralized with 0.15 M NaCl salt. Two-step minimization and four-step equilibration with decreasing degrees of positional restraints as prescribed by CHARMM-GUI were employed to prepare the systems for production runs (108). Three independent 500 ns simulations were performed for each of the PulF-membrane systems. The temperature of the systems was maintained at 310 K by coupling to a Nosé-Hoover thermostat with a time constant of 1 ps (109, 110). The pressure of the systems was maintained at 1 atm using semi-isotropic coupling to a Parrinello-Rahman barostat with a time constant of 5 ps (111). The electrostatic interactions were calculated using the smooth particle mesh Ewald (PME) method with a real-space cut-off of 1.2 nm (112), while the van der Waals interactions were truncated at 1.2 nm with a force switch smoothing function applied between 1.0 and 1.2 nm. The LINCS algorithm was applied to constrain all covalent bonds involving hydrogen atoms, and a 2-fs integration time step was used (113). In another set of simulations, the PulF trimer structure generated by AF2 was simulated with an *E. coli* model membrane using the same protocols described above. All simulations were performed using GROMACS 2020 (114). Visualization was conducted using VMD (115), while pore analysis was performed using CHAP (116).

### Modeling of PulE:PulF complex

We used AlphaFold2 to predict models of the trimeric PulF (without the N-domain) complexed with hexameric PulE (without the first flexible N-terminal domain N1D). It was not possible to model a full-length PulE:PulF complex with 6:3 stoichiometry using AlphaFold2 since the memory required by the 4,185 residues exceeded the memory available on our GPU systems. In this AF2 prediction with templates, we used the “*multimer*” model parameters (v. 2.3) for prediction and scoring (43). For sake of comparison, we also generated models of hexameric PulE alone or in complex with a PulF dimer.

Comparative models of *K. oxytoca* PulE hexamers were obtained from the SWISS-MODEL platform (117) using the structure of the *V. cholerae* GspE hexamer with quasi-C_6_ symmetry or C2 symmetry as template (PDB id 4KSS & 4KSR, respectively) (48).

## SUPPLEMENTAL MATERIAL

Supplemental tables and figures mbio.01423-23-s0001.pdf Tables S1 and S2; Figures S1-S16.

ASM does not own the copyrights to Supplemental Material that may be linked to, or accessed through, an article. The authors have granted ASM a non-exclusive, world-wide license to publish the Supplemental Material files. Please contact the corresponding author directly for reuse.
