# Supplementary material for: Membrane platform protein PulF of the Klebsiella type II secretion system forms a trimeric ion channel essential for endopilus assembly and protein secretion
Source: mBio. 2023 Dec 8;15(1):e01423-23. doi: 10.1128/mbio.01423-23 (PMC10790770; doi:10.1128/mbio.01423-23)
Supplement: Supplemental tables and figures — Tables S1 and S2; Figures S1-S16. [file mbio.01423-23-s0001.pdf]

Supplemental data

for

# Membrane platform protein PulF of the *Klebsiella* type II secretion system forms a trimeric ion channel essential for endopilus assembly and protein secretion

Ingrid Guilvout[1], Firdaus Samsudin[2], Roland Huber[2], Peter J. Bond[2,3], Benjamin Bardiaux[4,5], Olivera Francetic[1]

[1]Biochemistry of Macromolecular Interactions Unit, Department of Structural Biology and Chemistry, CNRS UMR3528, Institut Pasteur, 75724 Paris, France
[2]Bioinformatics Institute (A-STAR), 30 Biopolis Str, #07-01 Matrix, Singapore 138671
[3]Department of Biological Sciences, National University of Singapore, Singapore 117543
[4]Institut Pasteur, Structural Bioinformatics Unit, CNRS UMR3528, Department of Structural Biology and Chemistry, CNRS UMR3528, Institut Pasteur, Paris, France.
[5]Institut Pasteur, Bacterial Transmembrane Systems Unit, CNRS UMR3528, Department of Structural Biology and Chemistry, CNRS UMR3528, Institut Pasteur, Paris, France

**This PDF file includes:**

Supplementary Tables 1 and 2
Supplementary Figures 1 to 16
Supplementary References

**Table S1. Plasmids used in this study.**

| Plasmid name | Ori/resistance | Relevant markers | Primers used[1] | Source/reference |
|---|---|---|---|---|
| pCHAP8185 | ColE1/Ap$^R$ | *pulS,pelBsp-pulA,pulB, pulCDEFHIJKLMNO* | | (1) |
| pSU18 | p15A/Cm$^R$ | *placZ-lacZ$\alpha$* | | (2) |
| pUC18 | ColE1/Ap$^R$ | *placZ-lacZ$\alpha$* | | (3) |
| pCHAP8252 | ColE1/Ap$^R$ | pCHAP8185 *pulF$\Delta$(195-274)* | | This study |
| pCHAP6600 | ColE1/Ap$^R$ | Bsu36I-HindIII fragment of pCHAP8185 | PF178-242/ PF250-181 | This study |
| pCHAP6601 | ColE1/Ap$^R$ | pCHAP8185 *pulF $\Delta$(1-54)* | | This study |
| pCHAP6541 | ColE1/Ap$^R$ | Bsu36I-NotI fragment of pCHAP8185 | PF178-173/180-181 | This study |
| pCHAP6539 | ColE1/Ap$^R$ | pCHAP8185 $\Delta$*pulF* | | This study |
| pCHAP6469 | p15A/Cm$^R$ | PulF 55-401 | PF78-F3’ | This study |
| pCHAP6650 | ColE1/Ap$^R$ | PulF 55-401 | PF159-160 | This study |
| pCHAP8259 | p15A/Cm$^R$ | *placZ pulF* | PFEcoL-PFXbaR | This study |
| pCHAP7802 | ColE1/Ap$^R$ | *placZ-pulF* | PFEcoL-PFXbaR | Javier Santos Moreno |
| pCHAP6528 | ColE1/Ap$^R$ | *placZ pulF M166C* | PF151-152 | This study |
| pCHAP6529 | ColE1/Ap$^R$ | *placZ pulF Y168C* | PF153-154 | This study |
| pCHAP6516 | ColE1/Ap$^R$ | *placZ pulF V171C* | PF127-128 | This study |
| pCHAP6517 | ColE1/Ap$^R$ | *placZ pulF L172C* | PF129-130 | This study |
| pCHAP6542 | ColE1/Ap$^R$ | *placZ pulF T173C* | PF131b-132b | This study |
| pCHAP6519 | ColE1/Ap$^R$ | *placZ pulF L174C* | PF133-134 | This study |

| | | | | |
|---|---|---|---|---|
| pCHAP6520 | ColE1/$Ap^R$ | *placZ pulF V175C* | PF135-136 | This study |
| pCHAP6521 | ColE1/$Ap^R$ | *placZ pulF A176C* | PF137-138 | This study |
| pCHAP6522 | ColE1/$Ap^R$ | *placZ pulF V177C* | PF139-140 | This study |
| pCHAP6523 | ColE1/$Ap^R$ | *placZ pulF S178C* | PF141-142 | This study |
| pCHAP6524 | ColE1/$Ap^R$ | *placZ pulF V181C* | PF143-144 | This study |
| pCHAP6525 | ColE1/$Ap^R$ | *placZ pulF L183C* | PF145-146 | This study |
| pCHAP6526 | ColE1/$Ap^R$ | *placZ pulF S185C* | PF147-148 | This study |
| pCHAP6527 | ColE1/$Ap^R$ | *placZ pulF V187C* | PF149-150 | This study |
| pCHAP6530 | ColE1/$Ap^R$ | *placZ pulF A219C* | PF155-156 | This study |
| pCHAP6531 | ColE1/$Ap^R$ | *placZ pulF L223C* | PF157-158 | This study |
| pCHAP6532 | ColE1/$Ap^R$ | *placZ pulF L224C* | PF159-160 | This study |
| pCHAP6533 | ColE1/$Ap^R$ | *placZ pulF L225C* | PF161-162 | This study |
| pCHAP6534 | ColE1/$Ap^R$ | *placZ pulF A226C* | PF163-164 | This study |
| pCHAP6535 | ColE1/$Ap^R$ | *placZ pulF L228C* | PF165-166 | This study |
| pCHAP6536 | ColE1/$Ap^R$ | *placZ pulF L229C* | PF167-168 | This study |
| pCHAP6537 | ColE1/$Ap^R$ | *placZ pulF I231C* | PF169-170 | This study |
| pCHAP6448 | ColE1/$Ap^R$ | *placZ pulF L364C* | PF37-38 | This study |
| pCHAP6439 | ColE1/$Ap^R$ | *placZ pulF L366C* | PF39-40 | This study |
| pCHAP6440 | ColE1/$Ap^R$ | *placZ pulF S367C* | PF41-42 | This study |
| pCHAP6441 | ColE1/$Ap^R$ | *placZ pulF L368C* | PF43-44 | This study |
| pCHAP6512 | ColE1/$Ap^R$ | *placZ pulF F369C* | PF119-120 | This study |
| pCHAP6442 | ColE1/$Ap^R$ | *placZ pulF E370C* | PF45-46 | This study |
| pCHAP6443 | ColE1/$Ap^R$ | *placZ pulF L372C* | PF47-48 | This study |
| pCHAP6449 | ColE1/$Ap^R$ | *placZ pulF L373C* | PF49-50 | This study |
| pCHAP6450 | ColE1/$Ap^R$ | *placZ pulF V374C* | PF51-52 | This study |
| pCHAP6444 | ColE1/$Ap^R$ | *placZ pulF V375C* | PF53-54 | This study |
| pCHAP6446 | ColE1/$Ap^R$ | *placZ pulF A376C* | PF55-56 | This study |
| pCHAP6451 | ColE1/$Ap^R$ | *placZ pulF M377C* | PF57-58 | This study |
| pCHAP6445 | ColE1/$Ap^R$ | *placZ pulF M380C* | PF59-60 | This study |
| pCHAP6447 | ColE1/$Ap^R$ | *placZ pulF L382C* | PF61-62 | This study |
| pCHAP6513 | ColE1/$Ap^R$ | *placZ pulF A387C* | PF121-122 | This study |
| pCHAP6514 | ColE1/$Ap^R$ | *placZ pulF L389C* | PF123-124 | This study |
| pCHAP6515 | ColE1/$Ap^R$ | *placZ pulF L393C* | PF125-126 | This study |
| pCHAP6566 | p15A/$Cm^R$ | *placZ pulF M166C* | PF151-152 | This study |
| pCHAP6567 | p15A/$Cm^R$ | *placZ pulF Y168C* | PF153-154 | This study |
| pCHAP6662 | p15A/$Cm^R$ | *placZ pulF V171C* | PF127-128 | This study |
| pCHAP6663 | p15A/$Cm^R$ | *placZ pulF L172C* | PF129-130 | This study |
| pCHAP6664 | p15A/$Cm^R$ | *placZ pulF T173C* | PF131b-132b | This study |
| pCHAP6665 | p15A/$Cm^R$ | *placZ pulF L174C* | PF133-134 | This study |
| pCHAP6666 | p15A/$Cm^R$ | *placZ pulF V175C* | PF135-136 | This study |
| pCHAP6667 | p15A/$Cm^R$ | *placZ pulF A176C* | PF137-138 | This study |
| pCHAP6668 | p15A/$Cm^R$ | *placZ pulF V177C* | PF139-140 | This study |
| pCHAP6669 | p15A/$Cm^R$ | *placZ pulF S178C* | PF141-142 | This study |
| pCHAP6568 | p15A/$Cm^R$ | *placZ pulF V181C* | PF143-144 | This study |
| pCHAP6569 | p15A/$Cm^R$ | *placZ pulF L183C* | PF145-146 | This study |
| pCHAP6570 | p15A/$Cm^R$ | *placZ pulF S185C* | PF147-148 | This study |
| pCHAP6571 | p15A/$Cm^R$ | *placZ pulF V187C* | PF149-150 | This study |
| pCHAP6572 | p15A/$Cm^R$ | *placZ pulF A219C* | PF155-156 | This study |
| pCHAP6573 | p15A/$Cm^R$ | *placZ pulF L223C* | PF157-158 | This study |
| pCHAP6574 | p15A/$Cm^R$ | *placZ pulF L224C* | PF159-160 | This study |
| pCHAP6575 | p15A/$Cm^R$ | *placZ pulF L225C* | PF161-162 | This study |
| pCHAP6576 | p15A/$Cm^R$ | *placZ pulF A226C* | PF163-164 | This study |
| pCHAP6655 | p15A/$Cm^R$ | *placZ pulF L228C* | PF165-166 | This study |
| pCHAP6656 | p15A/$Cm^R$ | *placZ pulF L229C* | PF167-168 | This study |
| pCHAP6657 | p15A/$Cm^R$ | *placZ pulF I231C* | PF169-170 | This study |
| pCHAP6463 | p15A/$Cm^R$ | *placZ pulF L364C* | PF37-38 | This study |
| pCHAP6454 | p15A/$Cm^R$ | *placZ pulF L366C* | PF39-40 | This study |
| pCHAP6455 | p15A/$Cm^R$ | *placZ pulF S367C* | PF41-42 | This study |
| pCHAP6456 | p15A/$Cm^R$ | *placZ pulF L368C* | PF43-44 | This study |
| pCHAP6658 | p15A/$Cm^R$ | *placZ pulF F369C* | PF119-120 | This study |

| | | | | |
|---|---|---|---|---|
| pCHAP6457 | p15A/$Cm^R$ | *placZ pulF E370C* | PF45-46 | This study |
| pCHAP6458 | p15A/$Cm^R$ | *placZ pulF L372C* | PF47-48 | This study |
| pCHAP6464 | p15A/$Cm^R$ | *placZ pulF L373C* | PF49-50 | This study |
| pCHAP6465 | p15A/$Cm^R$ | *placZ pulF V374C* | PF51-52 | This study |
| pCHAP6459 | p15A/$Cm^R$ | *placZ pulF V375C* | PF53-54 | This study |
| pCHAP6461 | p15A/$Cm^R$ | *placZ pulF A376C* | PF55-56 | This study |
| pCHAP6466 | p15A/$Cm^R$ | *placZ pulF M377C* | PF57-58 | This study |
| pCHAP6460 | p15A/$Cm^R$ | *placZ pulF M380C* | PF59-60 | This study |
| pCHAP6462 | p15A/$Cm^R$ | *placZ pulF L382C* | PF61-62 | This study |
| pCHAP6659 | p15A/$Cm^R$ | *placZ pulF A387C* | PF121-122 | This study |
| pCHAP6660 | p15A/$Cm^R$ | *placZ pulF L389C* | PF123-124 | This study |
| pCHAP6661 | p15A/$Cm^R$ | *placZ pulF L393C* | PF125-126 | This study |
| pMS1264 | p15A/$Cm^R$ | *placZ pulF V171C P371C* | PF127-128/ PF295-296 | This study |
| pCHAP6728 | p15A/$Cm^R$ | *placZ pulF Y168C E370C* | PF45-46/ PF153-154 | This study |
| pUT18C | ColE1/$Ap^R$ | *placUV5-cyaA*T18 fragment in pUC18 | | (4)[9] |
| pKT25 | p15A/$Km^R$ | *placUV5-cyaA*T25 fragment in pSU38 | | (4) |
| pUT18C-ZIP | ColE1/$Ap^R$ | *placUV5-cyaA*T18-yeast Leu zipper | | (4) |
| pKT25-ZIP | p15A/$Km^R$ | *placUV5-cyaA*T25 yeast Leu zipper | | (4) |
| pCHAP8364 | ColE1/$Ap^R$ | T18-PulF | | (5) |
| pCHAP8365 | p15A/$Km^R$ | T25-PulF | | (5) |
| pCHAP6435 | ColE1/$Ap^R$ | T18-PulF 56-401 (ΔN) | PF35-36 | This study |
| pCHAP6434 | p15A/$Km^R$ | T25- PulF 56-401 (ΔN) | PF35-36 | This study |

[1]Where applicable

**Table S2. Oligonucleotides used in this study.**

| Primer name[1] | Sequence |
|---|---|
| PF EcoL | CAAGAATTCCGCGGAGGCTTGCTGAAATG |
| PF XbaR | CCCTCTAGACAATCACATACTCATCAAGG |
| PF35 | caaggtaccGCGCCGCACCAGCGC |
| PF36 | CCCGAATTCTCACATACTCATCAAGGTATTGAGC |
| PF78 | aagaattcCGCGGAGGCTTGCTGAAATGCGCCGCACCAGC |
| PF173 | GACAGCGCGGATTCACCTTAC |
| PF178 | ttagaattcACGATATTTATCTAGCCCTGAGGG |
| PF242 | tTCAGCAAGCCTCCGCGATCGGCTGC |
| PF250 | ATGCGCCGCACCAGCGCCCGCGATCTCGCC |
| PF181 | taaagcttCGGCAGCGTCTGCGCCTGCTGC |
| PF151-152 | CGCCTGCTGCAGGCGtgtATTTACCCCATCGTGC |
| PF178 | ttagaattcACGATATTTATCTAGCCCTGAGGG |
| PF242 | tTCAGCAAGCCTCCGCGATCGGCTGC |
| PF250 | ATGCGCCGCACCAGCGCCCGCGATCTCGCC |
| PF181 | taaagcttCGGCAGCGTCTGCGCCTGCTGC |
| PF153-154 | CTGCAGGCGATGATTtgtCCCATCGTGCTGACC |
| PF127-128 | CGATGATTTACCCCATCtgtCTGACCCTGGTGGC |
| PF129-130 | GATTTACCCCATCGTGtgtACCCTGGTGGCGGTC |
| PF131b-132b | GATTTACCCCATCGTGCTGtgtCTGGTGGCGGTCAGC |
| PF133-134 | CCCATCGTGCTGACCtgtGTGGCGGTCAGCGTG |
| PF135-136 | CCATCGTGCTGACCCTGtgtGCGGTCAGCGTGATCG |
| PF137-138 | CGTGCTGACCCTGGTGtgtGTCAGCGTGATCGTC |
| PF139-140 | CTGACCCTGGTGGCGtgtAGCGTGATCGTCATTC |
| PF141-142 | CCCTGGTGGCGGTCtgtGTGATCGTCATTCTGC |
| PF143-144 | GCGGTCAGCGTGATCtgtATTCTGCTGTCGACGG |
| PF145-146 | CAGCGTGATCGTCATTtgtCTGTCGACGGTGGTGC |
| PF147-148 | GATCGTCATTCTGCTGtgtACGGTGGTGCCGAAGG |
| PF149-150 | CATTCTGCTGTCGACGtgtGTGCCGAAGGTCGTCG |
| PF155-156 | CGATATGCTGCGCGCCtgtGGGCCGTGGCTGCTGC |
| PF157-158 | CCGCCGGGCCGTGGtgtCTGCTGGCGATTTTGCTCC |
| PF159-160 | GCCGGGCCGTGGCTGtgtCTGGCGATTTTGCTCC |
| PF161-162 | GGGCCGTGGCTGCTGtgtGCGATTTTGCTCCTC |
| PF163-164 | CCGTGGCTGCTGCTGtgtATTTTGCTCCTCATTCTC |
| PF165-166 | GCTGCTGCTGGCGATTtgtCTCCTCATTCTCCTGC |
| PF167-168 | GCTGCTGGCGATTTTGtgtCTCATTCTCCTGCTGCG |
| PF169-170 | GGCGATTTTGCTCCTCtgtCTCCTGCTGCGCTACC |
| PF37-38 | GAGCGCACAAATTCAGtgtGCCCTCAGCCTGTTTG |
| PF39-40 | CAAATTCAGCTGGCCtgtAGCCTGTTTGAACCCC |
| PF41-42 | CAAATTCAGCTGGCCCTCtgtCTGTTTGAACCCCTGC |
| PF43-44 | CAGCTGGCCCTCAGCtgtTTTGAACCCCTGCTG |
| PF119-120 | GCTGGCCCTCAGCCTGTgTGAACCCCTGCTGGTGG |
| PF45-46 | GCCCTCAGCCTGTTTtgtCCCCTGCTGGTGGTG |
| PF47-48 | CTCAGCCTGTTTGAACCCtgtCTGGTGGTGGCGATGGCC |
| PF49-50 | CCTGTTTGAACCCCTGtgtGTGGTGGCGATGGCCG |
| PF51-52 | GTTTGAACCCCTGCTGtgtGTGGCGATGGCCGGC |
| PF53-54 | GAACCCCTGCTGGTGtgtGCGATGGCCGGCATG |
| PF55-56 | CCCCTGCTGGTGGTGtgtATGGCCGGCATGGTG |
| PF57-58 | CTGCTGGTGGTGGCGtgtGCCGGCATGGTGCTG |
| PF59-60 | GTGGCGATGGCCGGCtgtGTGCTGTTTATCGTC |
| PF61-62 | GATGGCCGGCATGGTGtgtTTTATCGTCCTCGCC |
| PF121-122 | GCTGTTTATCGTCCTCtgtATCCTGCAGCCGATACTGC |
| PF123-124 | GTTTATCGTCCTCGCCATCtgtCAGCCGATACTGCAGC |
| PF125-126 | CCATCCTGCAGCCGATAtgtCAGCTCAATACCTTG |
| PF295-296 | CCTCAGCCTGTTTGAAtgtCTGCTGGTGGTGGCG |

[1] The second primer in a pair, where indicated, is the reverse complement of the first one.

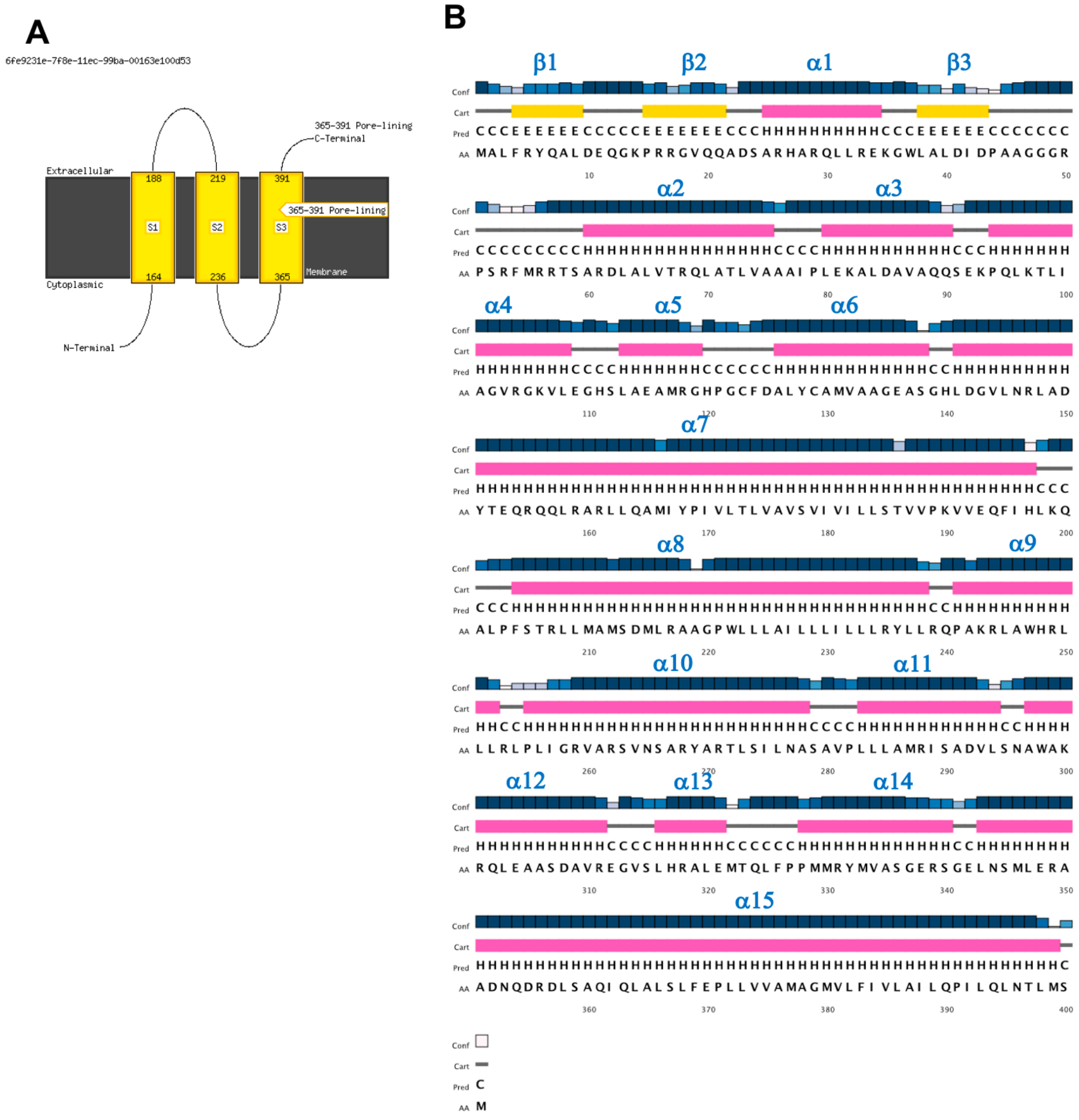


**Figure S1. The PulF topology and secondary structure predictions generated by the PSIPRED server (http://bioinfadmin.cs.ucl.ac.uk/). A**. MEMSAT_SVM prediction of PulF topology and boundaries of the three transmembrane segments. TMS3 is predicted as a pore-lining helix. **B.** The PsiPred (6) prediction showing PulF primary sequence, with α-helix (pink rectangles), β-sheet (yellow rectangles) and loop (black lines) regions indicated. The α-helix and β-sheet secondary structure elements are numbered and indicated in blue. Per-residue prediction confidence levels, ranging from low (white) to high confidence (dark blue), are shown above the protein sequence.

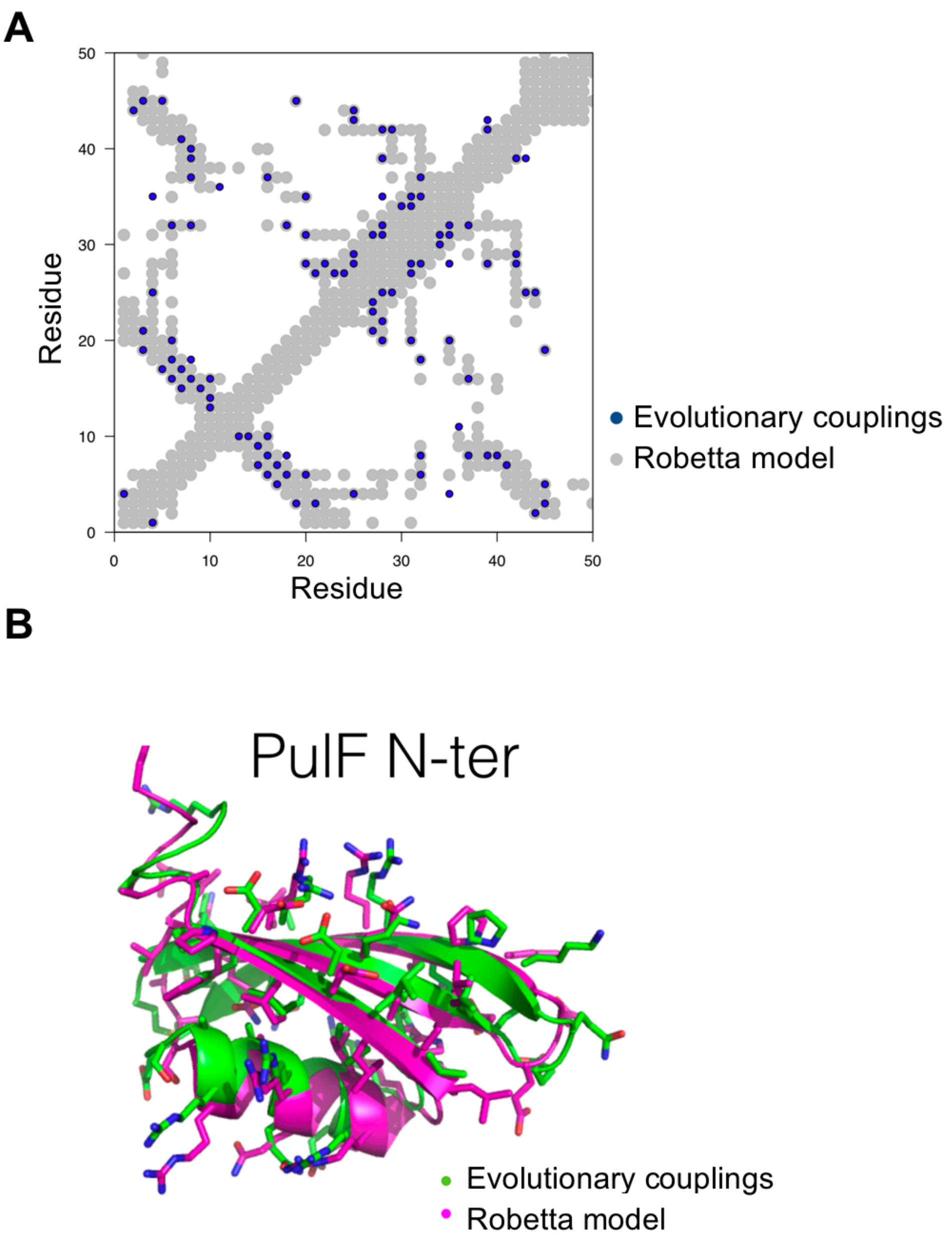


**Figure S2**. **Modeling of the PulF N-domain. A.** Pairwise residue contact map (cutoff < 6Å) from the N-terminal PulF model generated by Robetta (grey dots) and contact prediction from Gremlin (blue dots). **B**. Structural model of N-domain obtained from evolutionary couplings (green) superimposed with the Robetta model (magenta). Side chains are shown as licorice.

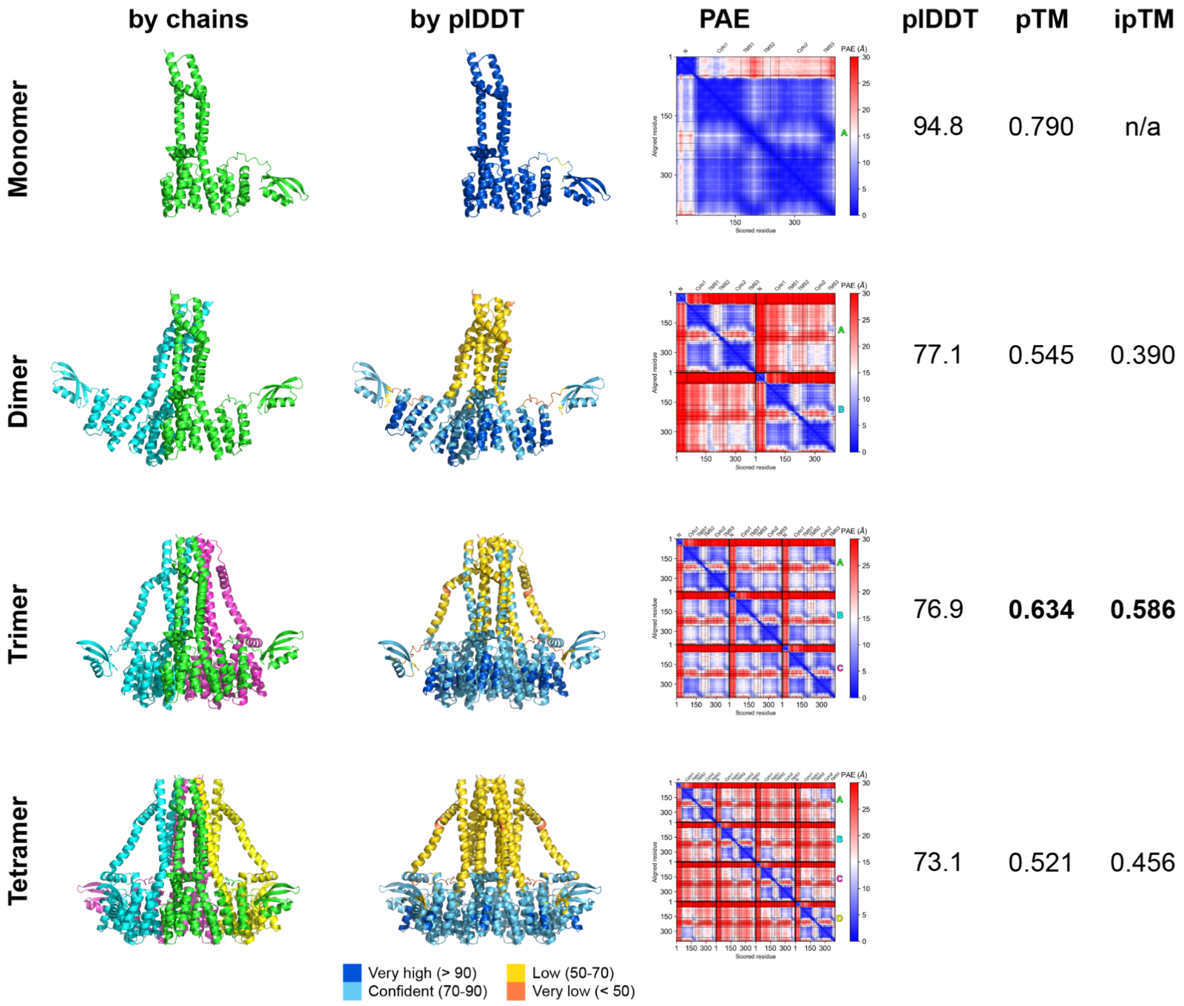


**Figure S3. AlphaFold2-multimer models and scores for PulF in different oligomeric states.** The best scoring (highest *multimer* score) models for monomeric, dimeric, trimeric and tetrameric (top to bottom rows) PulF are shown as cartoon and colored by chains (left column) or by predicted local Distance Difference Test values (plDDT, 2nd column from the left). The corresponding predicted aligned error maps (PAE), colored from blue (low PAE) to red (high PAE) are presented in the 3rd column from the left. Each chain block is labeled by the chain id with the corresponding color as shown on the left column. The last three columns on the right report the various confidence scores from AF2: average plDDT, predicted TM-score (pTM) and Interface pTM (ipTM). The trimer PulF model exhibits the highest pTM and ipTM scores and the lowest inter-chain PAE.

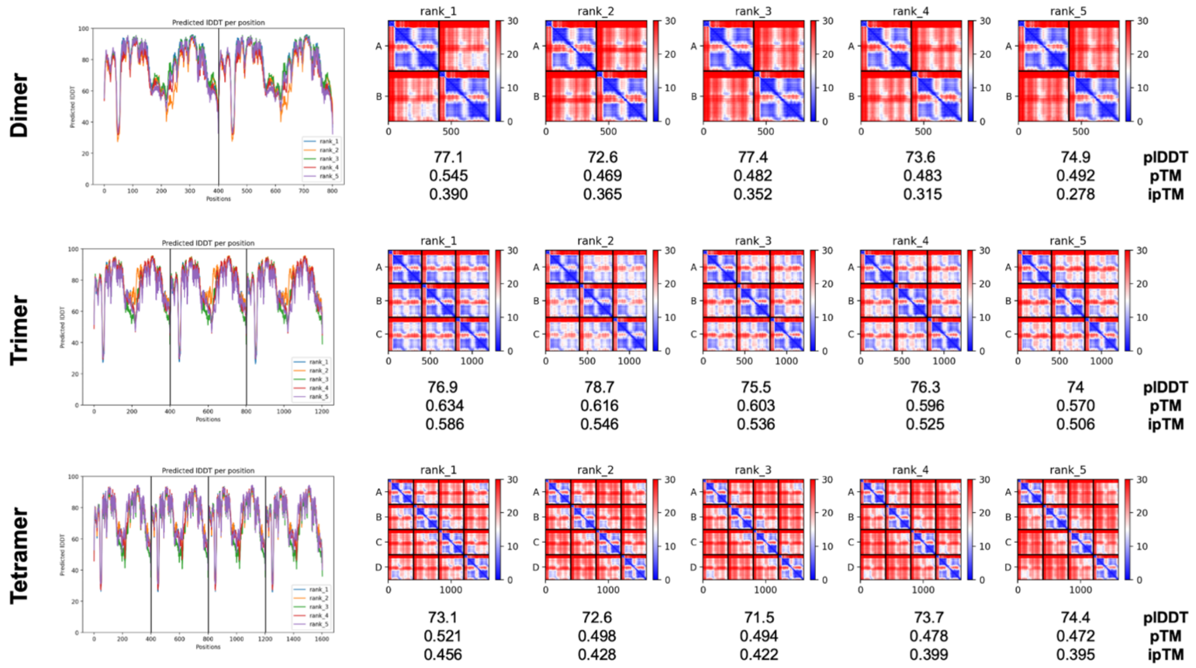


**Figure S4. AlphaFold2-multimer scores for the five models of PulF in different oligomeric states.** Left column: Predicted local Distance Difference Test (pIDDT) values along the PulF sequence for the five ranked models (the different chains in the oligomer are concatenated). Right columns: predicted aligned error (PAE) maps for the five ranked models and colored from blue (low PAE) to red (high PAE). Below are reported the average pIDDT, pTM and ipTM scores for each model. The ipTM values for all trimeric PulF models are higher than the best scoring dimeric or tetrameric models.

A

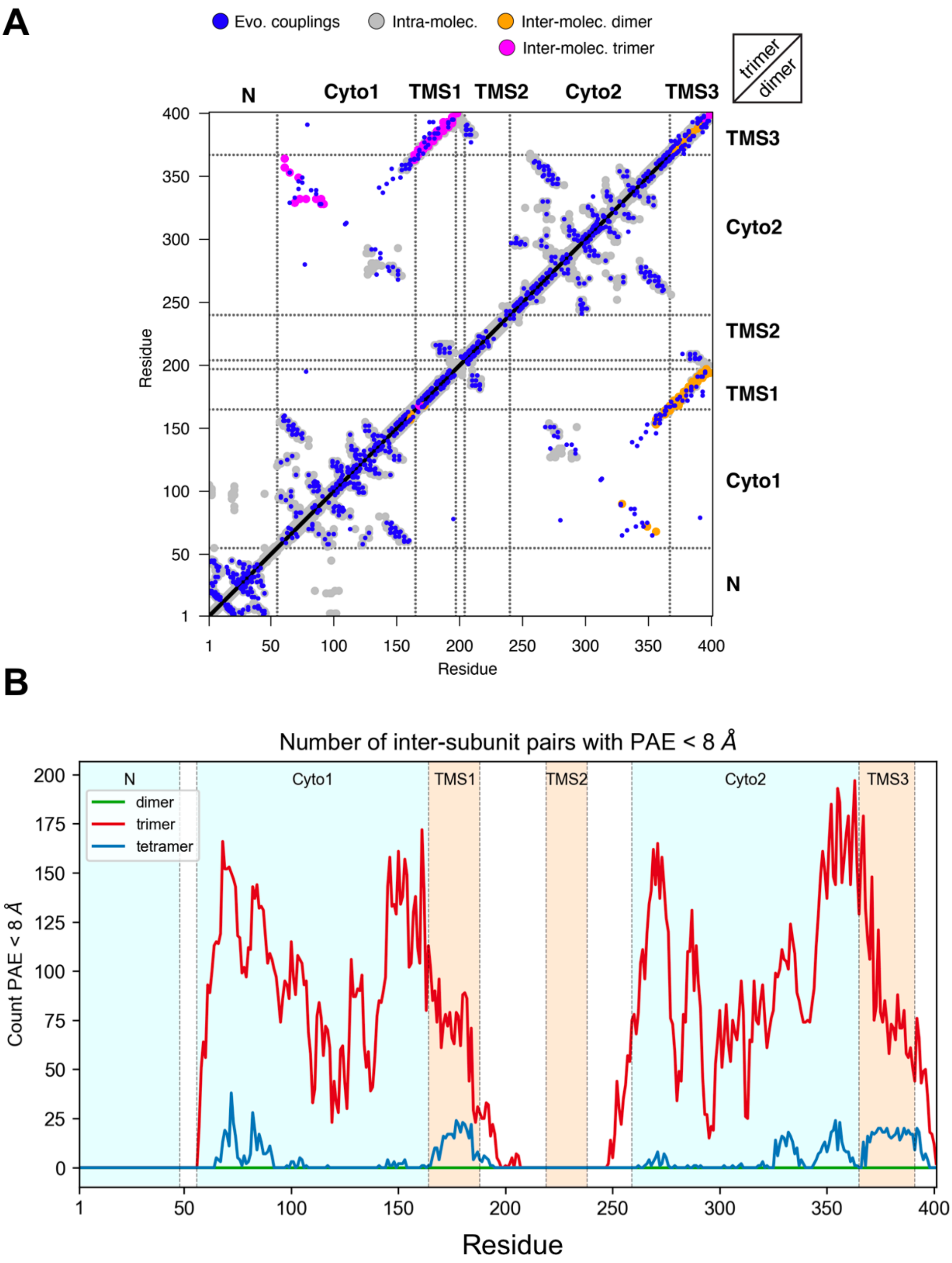


**Figure S5. Comparison of oligomeric PulF models predicted by AlphaFold2-multimer. A**. Contact maps of dimeric (lower-triangle) and trimeric (upper-triangle) PulF models. Observed intra-molecular contacts are colored in grey and inter-molecular contacts are colored in orange and magenta for the dimeric and trimeric models, respectively. Predicted evolutionary couplings are colored in dark blue. The overlap between predicted evolutionary contacts and inter-molecular contacts is higher for the PulF trimer model. **B.** Amount of inter-chain residue pairs with a predicted aligned error (PAE) < 8 Å along the PulF sequence in the dimeric, trimeric and tetrameric PulF models. Positions of PulF domains are indicated on top. The trimeric PulF model displays a significantly higher number of high-confidence inter-chain contacts compared to the dimeric and tetrameric models.

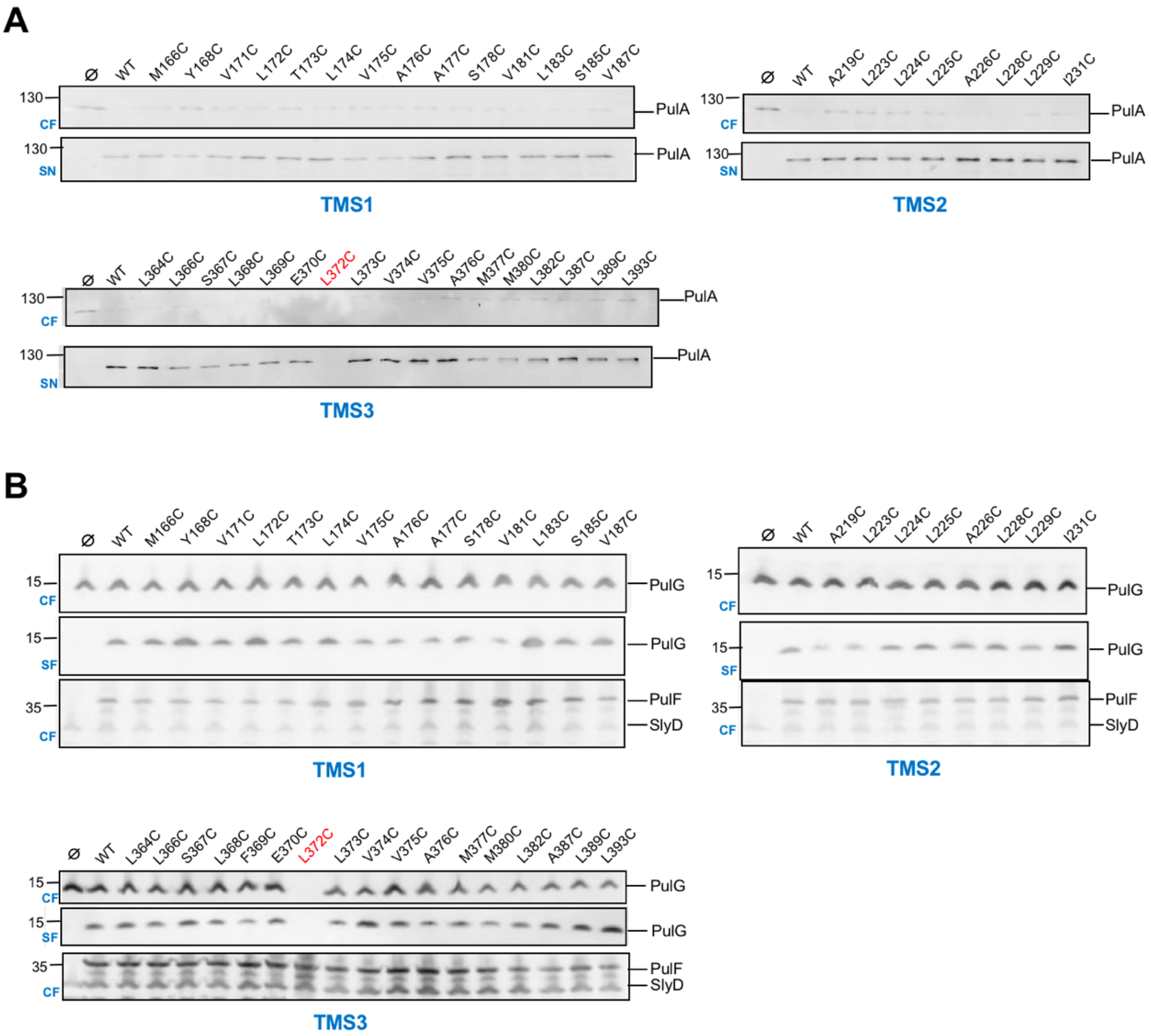


**Figure S6. The PulF Cys substituted variants are functional. A.** PulA secretion promoted by PulF variants with single Cys substitutions in PulF TM segments as indicated. Secretion of PulA was assayed in *E. coli* strain PAP5207 mimicking the chromosomal expression levels of *pul* genes. Cell and supernatant (SN) fractions from an equivalent of 0.1 $OD_{600nm}$ of bacteria were analyzed by SDS-PAGE and Western blot with anti-PulA antibodies. **B.** Assembly of PulG pili promoted by indicated PulF variants in strain PAP7460 strain carrying the *pul* genes on plasmid pCHAP8252 complemented with pCHAP8259 derivatives carrying wild type or mutant *pulF* as indicated. Cell fractions (CF) and sheared fractions (SF) from an equivalent of 0.1 $OD_{600nm}$ of bacteria were analyzed by SDS-PAGE and Western blot with anti-PulG antibodies. Cell fractions were further probed by anti-$PulF_N$ antibodies and PulF protein levels are shown at the bottom of each panel. SlyD indicates the cross-reacting band revealed by the anti-PulF sera. Relevant Mw markers (in kDa) are indicated on the left. The PulF L372C variant labeled in red caused degradation of PulA and PulG.

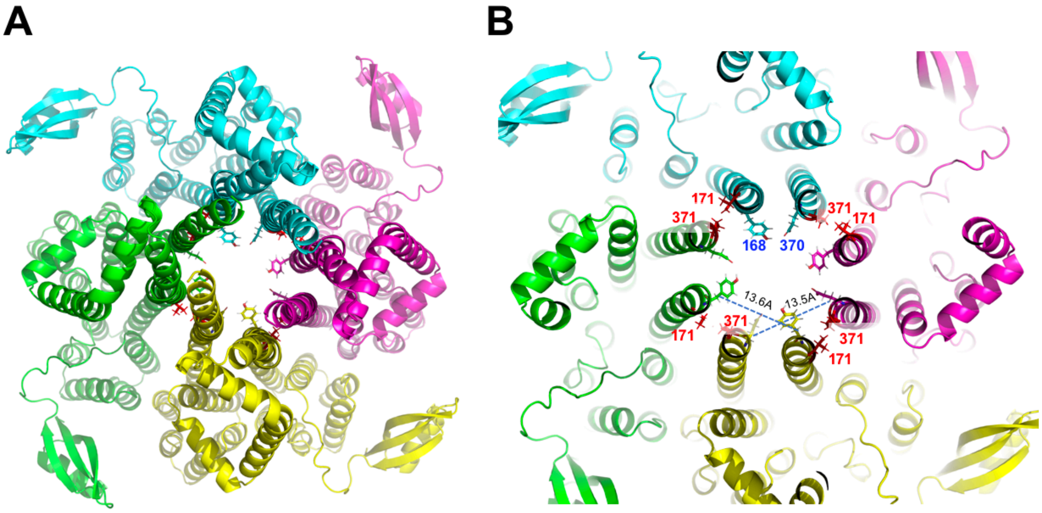


**Figure S7. The PulF tetramer model is incompatible with the cross-linking data. A.** The cartoon representation of the PulF tetramer model (top view) generated by AF2 algorithm, with monomers highlighted in different colors. The side chains of residues 168, 171, 370 and 371 are shown as sticks. **B.** A zoom on the PulF tetramer model showing the channel residues 168 and 370 (in blue), as well as interface residues 171 and 371 (in red), in stick representation. The distance between 171 and 371 side chains is compatible with crosslinking, similar to the trimer model; however, no tetrameric forms of PulF were detected upon crosslinking of variant PulF$^{V171C-P371C}$, arguing against this model. The distance between the C$\beta$ atoms of the channel residues Y168 and E370 in the tetramer model is too large and is thus incompatible with crosslinking of dimers and trimers.

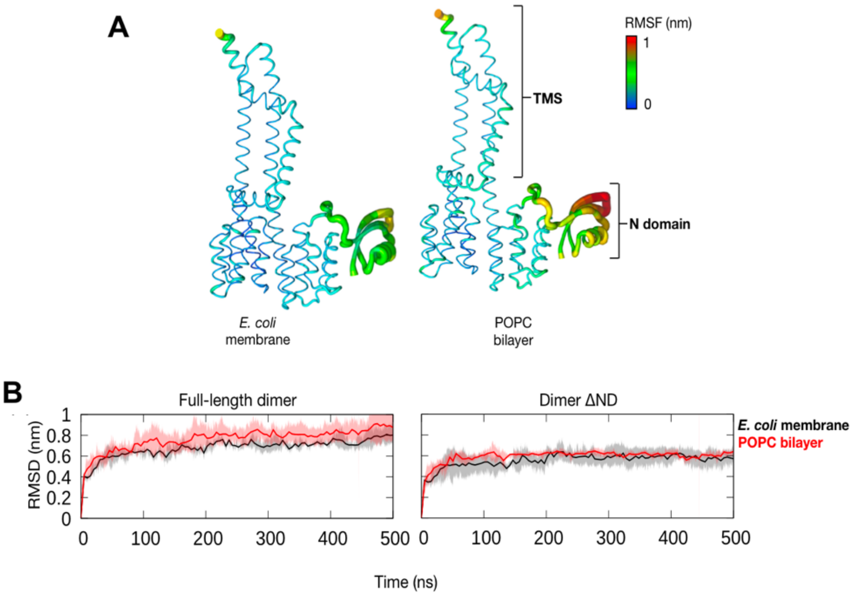


Figure S8. **Comparison of protein dynamics between simulations in *E. coli* membrane versus pure POPC bilayer. A.** Per-residue RMSF values mapped on to the structure of the protein showing a more mobile N-domain in simulations with POPC bilayer. **B.** Backbone RMSD for full-length PulF (left) or PulF variant lacking the N-domain ($\Delta$ND, right), highlighting the increased flexibility of the N-domain in simulations with POPC bilayer. Solid lines show average values from three simulations, and standard deviations are illustrated by the shaded areas.

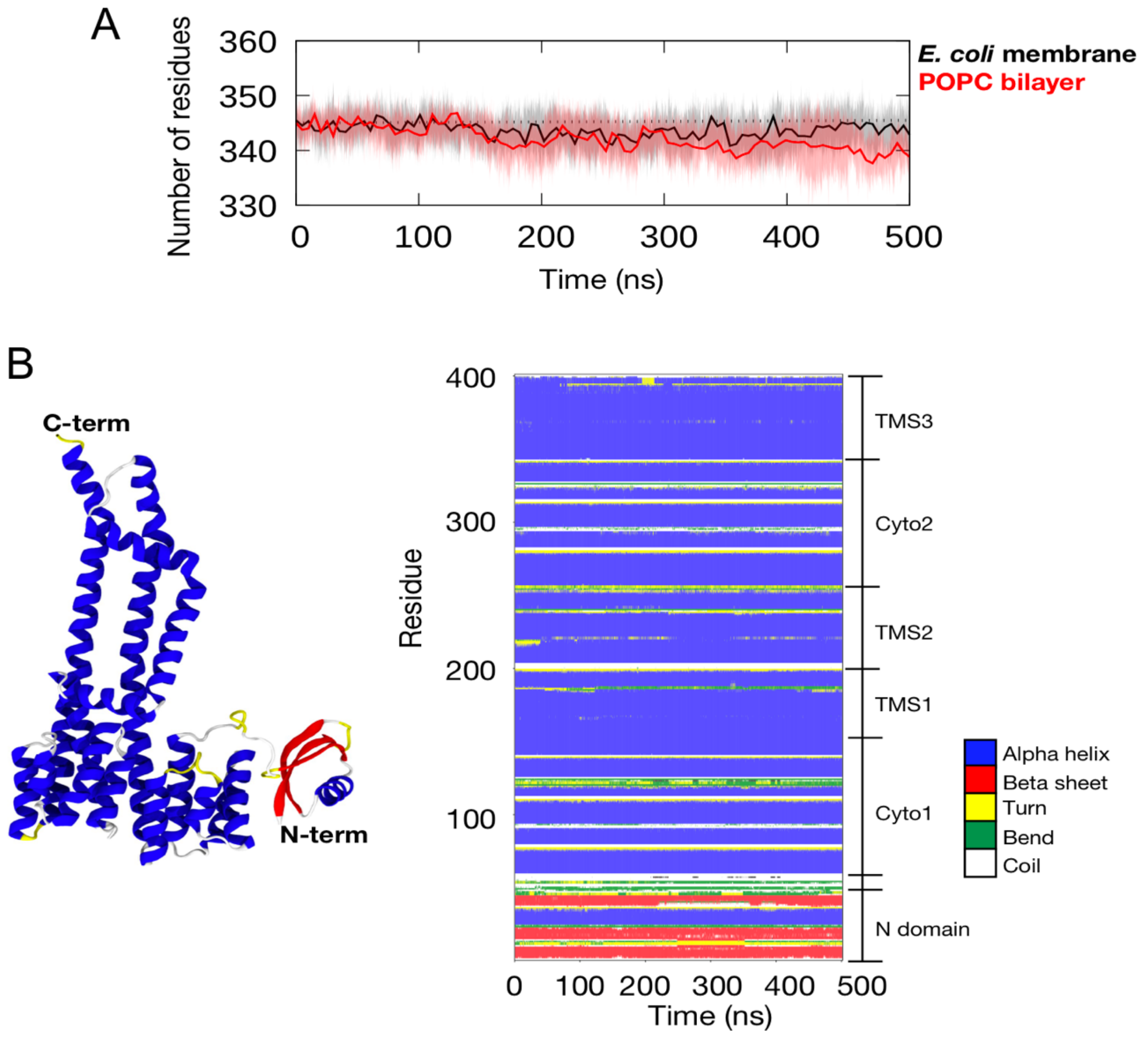


**Figure S9. Secondary structure preservation during MD simulations. A**. The total number of residues with secondary structural elements (α-helix, β-sheet, turn and bend) throughout the trajectories in an *E. coli* membrane model (black) and a POPC bilayer (red). Solid lines show average over two monomers and three simulations, while shaded areas indicate standard deviations. Dotted line indicates the number of residues with secondary structure predicted by AlphaFold2. **B**. PulF monomer colored based on the secondary structure (left) and the conservation of the secondary structure throughout a simulation in an *E. coli* membrane model (right).

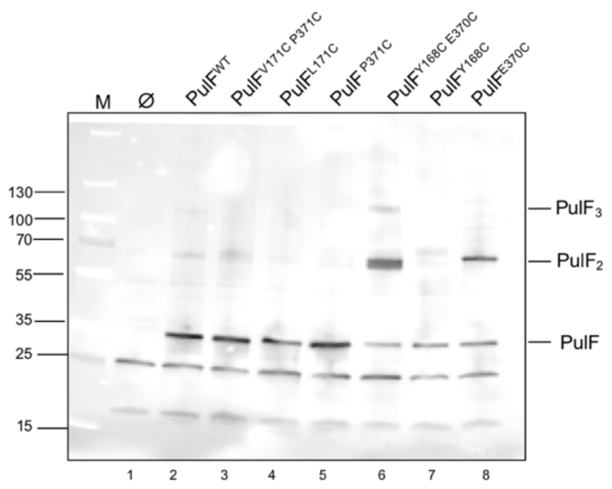


**Figure S10. Spontaneous oxidation of Cys residues in total bacterial extracts.** Bacteria of strain PAP7460 containing plasmid pCHAP8252 and plasmid pCHAP8259 or its derivatives were cultured as indicated in Fig. 9 of the Main text. Total extracts were analyzed on 4-15 % SDS gradient gels and Western blot with anti-PulF$_N$ antibodies. The migration of PulF monomers, dimers and trimers are indicated on the right.

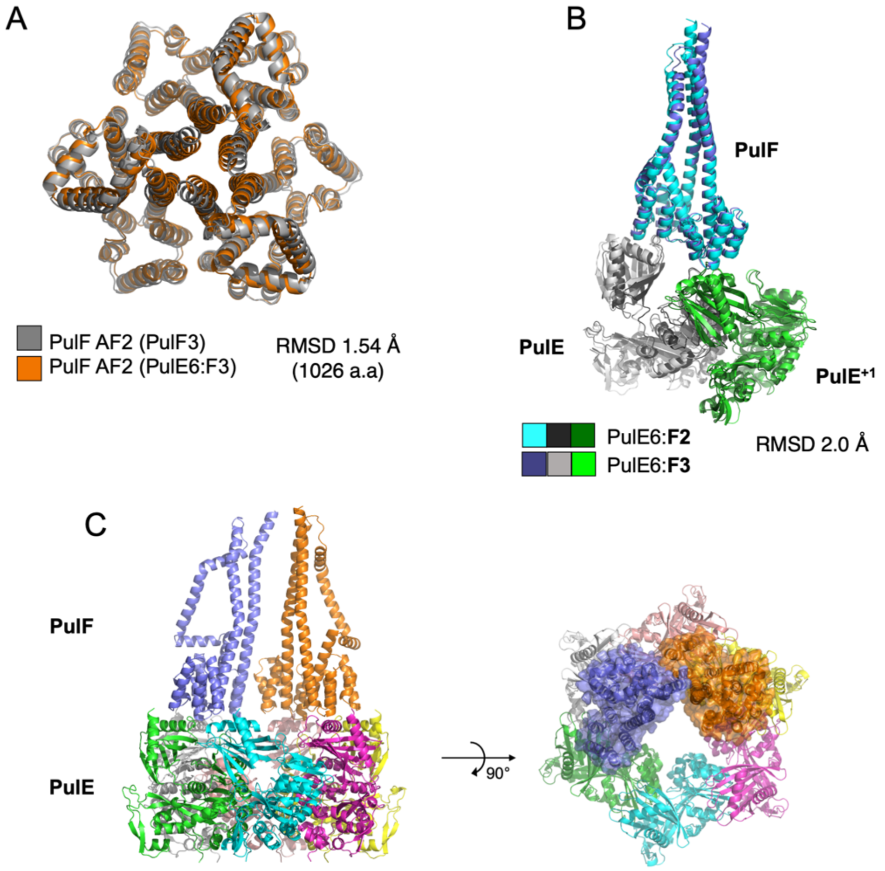


**Figure S11. Comparison of PulF structures predicted with AF2-multimer**. **A.** Superposition of AF2 models for the PulF trimer alone (PulF3, grey) or in complex with PulE (PulE6:F3, orange). The two structures are virtually identical with an RMSD of 1.5 Å for all residues. **B.** Superposition of a PulE2:PulF sub-complex from PulE:PulF complex models predicted by AF2 with either dimeric (PulE6:**F2**) or trimeric (PulE6:**F3**) PulF. The PulF subunit structure is nearly identical as is the orientation of the PulE subunits and the PulE-PulF interface between the Cyto1/2 domains and neighboring N2D domains (overall RMSD 2.0 Å). **C.** Model of a PulE:PulF complex predicted with AF2-multimer with 6:2 stoichiometry and colored by chains. The two PulF subunits are colored in lilac and orange. The left view is rotated by 90° showing the two PulE subunits not interacting with PulF (cyan and magenta), a space occupied by a 3rd PulF subunit in the predicted PulE:PulF complex with 6:3 stoichiometry.

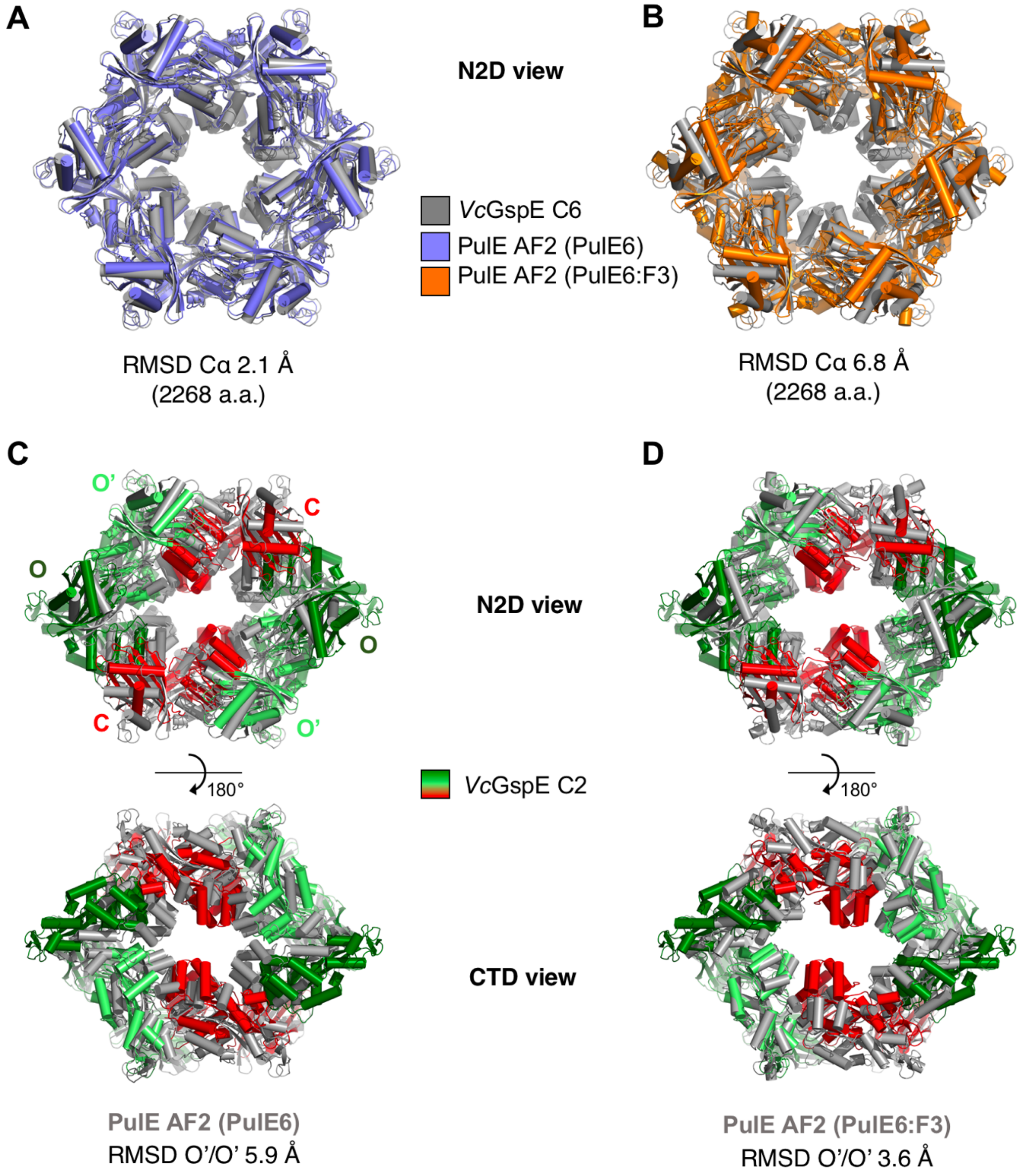


**Figure S12. Comparison of PulE hexameric structures predicted with AF2-multimer**. **A.** Superposition of AF2 PulE hexamer model (lilac) with *Vc*GspE structure with C6 symmetry (grey). **B.** Superposition of AF2 PulE hexamer from the $PulE_6$:$PulF_3$ model (orange) with *Vc*GspE structure with C6 symmetry (grey). When predicted alone, the PulE hexameric structure (PulE6) is closer (RMSD 2.1 Å) to the *Vc*GspE C6 structure than when modeled as a complex with trimeric PulF (PulE6:F3, RMSD 6.8 Å). The view is oriented with the N2D in front. **C.** Superposition of AF2 PulE hexamer model (PulE6, grey) with *Vc*GspE structure with C2 symmetry, in which the open, open' and closed subunits are colored in dark green, light green and red, respectively. The bottom view is rotated 180° showing the CTD in front. **D.** Same as **C** but superposing the AF2 PulE hexamer from the $PulE_6$:$PulF_3$ model (PulE6:F3, grey). The minimum RMSD between opposite subunits (same state) is found for the open' subunits in the $PulE_6$:$PulF_3$ model (3.6 Å).

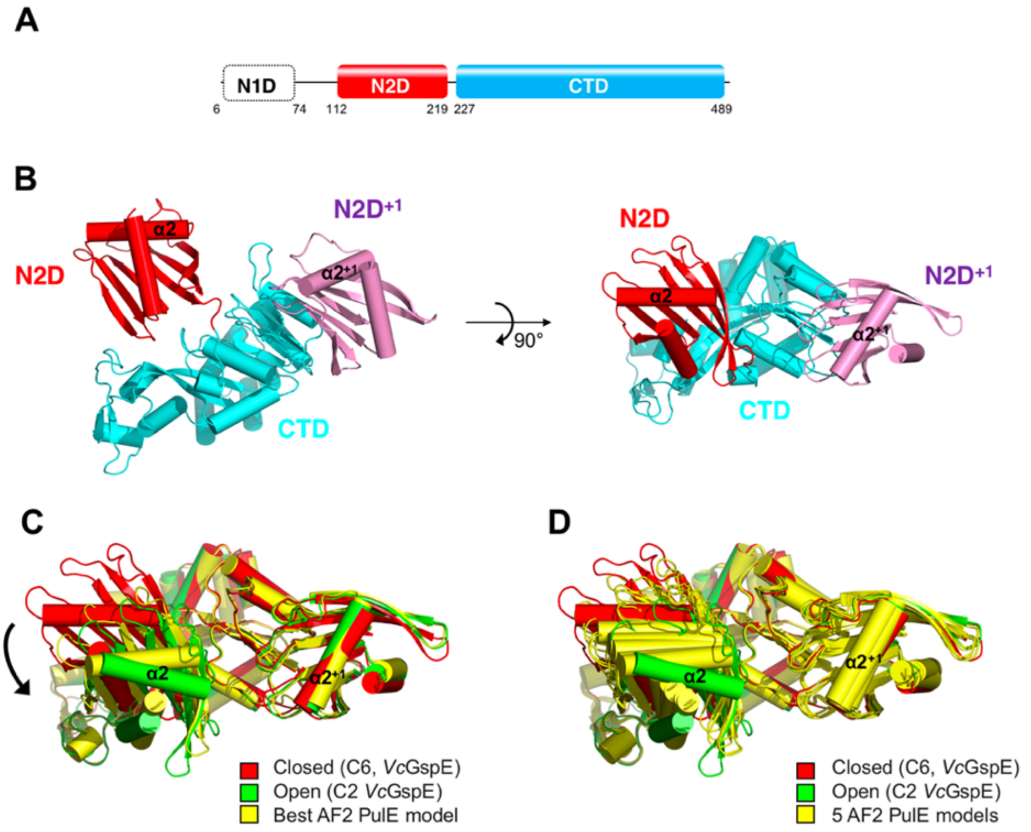


**Figure S13. Domain orientation in PulE/GspE hexamers**. **A.** PulE domain organization. The flexible domain N1 is shown in white, the N2 in red and CTD in cyan. **B.** Two domains are shown for each GspE/PulE subunit: N2D (red) and CTD (cyan). The CTD of one subunit interacts with the N2D of the neighboring subunits ($N2D^{+1}$, pink). The construction block formed by CTD:$N2D^{+1}$ is pseudo-rigid while the orientation of N2D with regard to CTD is variable for a single subunit, resulting in open and closed states depending on the internal symmetry of the GspE/PulE hexameric structure (C6 or C2). The view on the right is rotated by 90°. The position of the α2 helix is labeled. **C.** Superposition of a PulE subunit from the best AF2 model of PulE:PulF complex with 6:3 stoichiometry (yellow) with closed (red) and open (green) subunits in the *Vibrio cholerae* (*Vc*) GspE hexameric structures with C6 symmetry (PDB id 4kss) and C2 symmetry PDB id 4ksr), respectively. The orientation of the N2D in the best AF2 PulE hexamer model is similar to the open state observed in *Vc*GspE with C2 symmetry. Only residues from the CTD were used for superposition. The view is oriented as in **B** (right panel). **D.** Same as **C**, but showing the PulE subunit in all five AF2-multimer models for $PulE_6$:$PulF_3$.

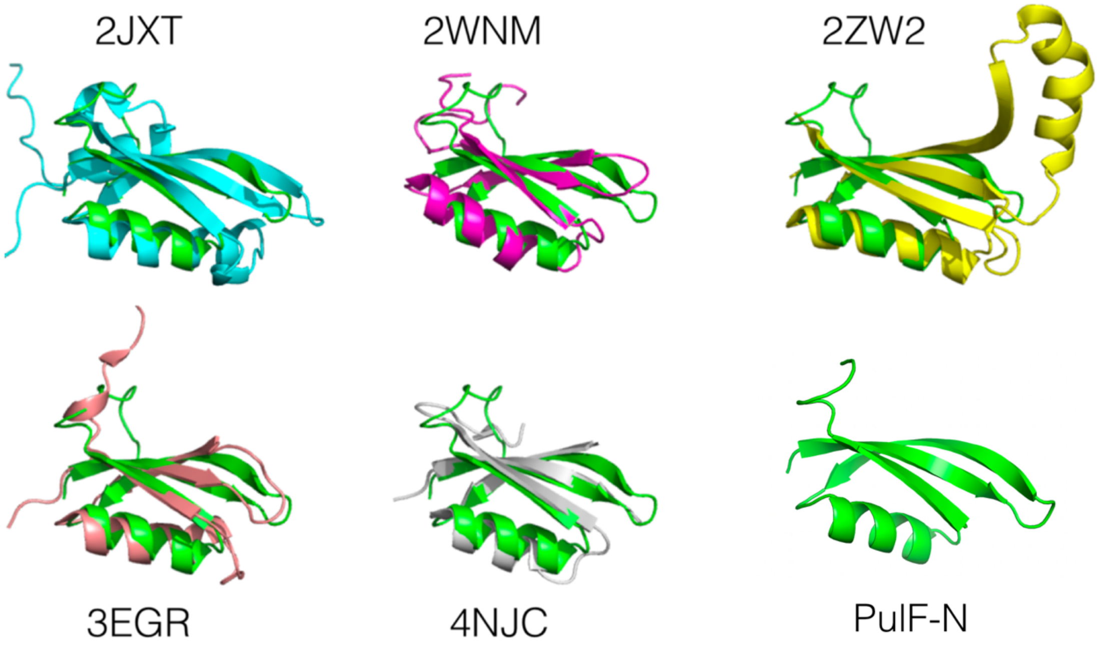


**Figure S14. The closest structural homologues of PulF N-domain superimposed with the PulF N-domain Robetta model (in green).** PDB accession codes are indicated for each image of superimposed structures: 2JXT (50S ribosomal protein LX from *Methanobacterium thermoautotrophicum,* unpublished); 2WNM (GP2 protein inhibitor of *E. coli* RNA polymerase from phage T7) (7); 2ZW2 (Formylglycinamide ribonucleotide amidotransferase III from *Sulfolobus tokodaii*, unpublished); 3EGR *Ralstonia eutropha* phenylacetate-CoA oxygenase subunit PaaB, unpublished) and 4NJC (RNA Polymerase interacting protein YkzG from *Geobacillus stearothermophilus*) (8).

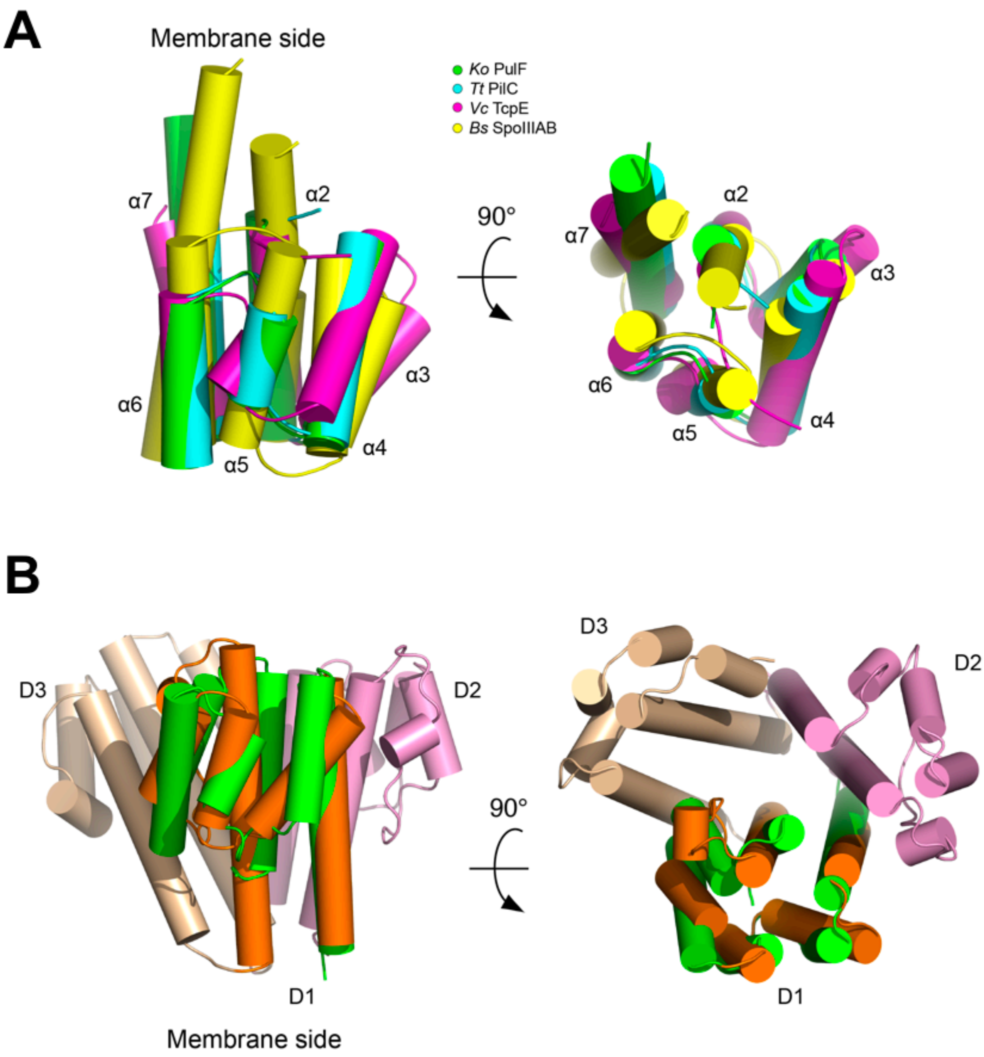


**Figure S15. The PulF Cyto1 structural homologues. A.** Homologs from the GspF/PilC family superimposed on the PulF Cyto1 model (green): N-terminal cytoplasmic domains of *T. thermophilus* PilC (cyan, PDB id 2WHN), *Vibrio cholerae* TcpE (magenta, PDB id 4HHX) and *Bacillus subtilis* SpoIIIAB (yellow, PDB id 6BS9). **B.** Superposition of PulF Cyto1 model onto the second domain of the d subunit from the *T. thermophilus* V-ATPase (PDB id 1R5Z). Each 6-helix domain of the V-ATPase D subunit is colored differently and PulF Cyto1 is shown in green. The orientation of the structures with regard to the membrane is indicated.

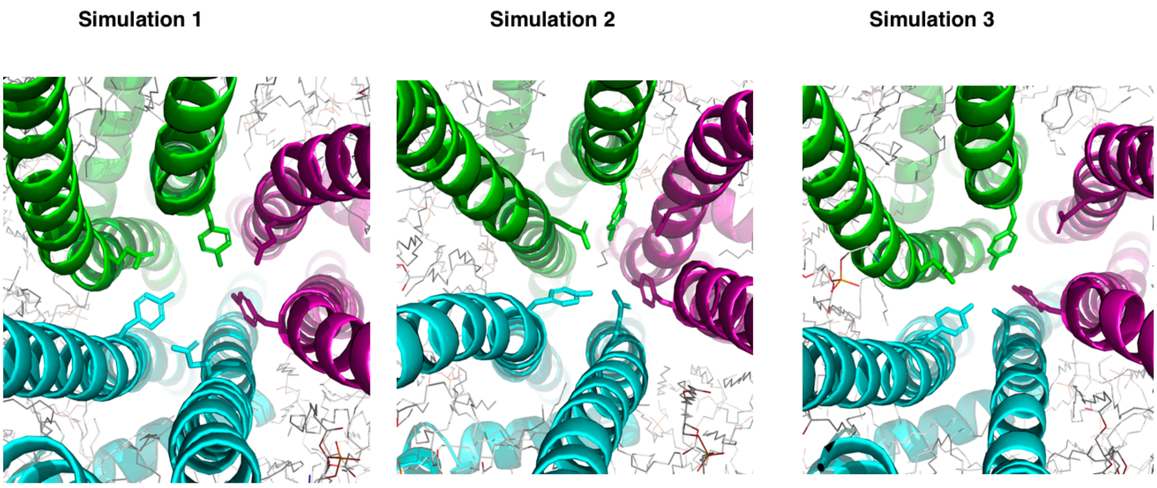


**Figure S16. The final snapshots of three PulF trimer simulations in *E. coli* membrane.** A top view is shown zooming on the polar channel residues. The protomers are shown in green, magenta and cyan with Y168 and E370 residues shown as sticks. Their positions deviate from the initial symmetric model changing the distance between the side chains and affecting the probability of direct contacts and crosslinking when replaced by Cys residues.

## Supplementary references


1. Cisneros DA, Bond PJ, Pugsley AP, Campos M, Francetic O. 2012. Minor pseudopilin self-assembly primes type II secretion pseudopilus elongation. The EMBO J 31:1041-1053.
2. Bartolomé B, Jubete Y, Martinez E, de la Cruz F. 1991. Construction and properties of a family of pACYC184-derived vectors compatible with pBR322 and its derivatives. Gene 102:75-78.
3. Yanisch-Perron C, Vieira J, Messing J. 1985. Improved M13 phage cloning vectors and host strains: nucleotide sequences of the M13mpl8 and pUC19 vectors. Gene 33:103-119.
4. Karimova G, Pidoux J, Ullmann A, Ladant D. 1998. A bacterial two-hybrid system based on a reconstituted signal transduction pathway. Proc Natl Acad Sci U S A 95:5752-5756.
5. Nivaskumar M, Santos-Moreno J, Malosse C, Nadeau N, Chamot-Rooke J, Tran Van Nhieu G, Francetic O. 2016. Pseudopilin residue E5 is essential for recruitment by the type 2 secretion system assembly platform. Mol Microbiol 101:924-941.
6. Buchan DWA, Jones DT. 2019. The PSIPRED Protein Analysis Workbench: 20 years on. Nucl Acids Res 47:W402-W407.
7. Cámara B, Liu M, Reynolds J, Shadrin A, Liu B, Kwok K, Simpson P, Weinzierl R, Severinov K, Cota E, Matthews S, Wigneshweraraj SR. 2010. T7 phage protein Gp2 inhibits the *Escherichia coli* RNA polymerase by antagonizing stable DNA strand separation near the transcription start site. Proc Natl Acad Sci USA 107:2247-2252.
8. Keller AN, Yang X, Wiedermannová J, Delumeau O, Krásný L, Lewis PJ. 2014. ε, a New Subunit of RNA Polymerase Found in Gram-Positive Bacteria. J Bacteriol 196:3622-3632.
